# Deriving an optimal threshold of waist circumference for detecting cardiometabolic risk in sub-Saharan Africa

**DOI:** 10.1038/ijo.2017.240

**Published:** 2017-10-31

**Authors:** K Ekoru, G A V Murphy, E H Young, H Delisle, C S Jerome, F Assah, B Longo–Mbenza, J P D Nzambi, J B K On'Kin, F Buntix, M C Muyer, D L Christensen, C S Wesseh, A Sabir, C Okafor, I D Gezawa, F Puepet, O Enang, T Raimi, E Ohwovoriole, O O Oladapo, P Bovet, W Mollentze, N Unwin, W K Gray, R Walker, K Agoudavi, S Siziya, J Chifamba, M Njelekela, C M Fourie, S Kruger, A E Schutte, C Walsh, D Gareta, A Kamali, J Seeley, S A Norris, N J Crowther, D Pillay, P Kaleebu, A A Motala, M S Sandhu

**Affiliations:** 1 Sandhu Group, Department of Medicine, University of Cambridge, Cambridge, UK; 2 Global Health and Populations Group, Wellcome Trust Sanger Institute, Hinxton, Cambridge, UK; 3 Centre for Tropical Medicine and Global Health, Nuffield Department of Medicine, University of Oxford, Oxford, UK; 4 Department of Nutrition, Faculty of Medicine, University of Montreal, Montreal, Canada; 5 Regional Institute of Public Health, University of Abomey-Calavi, Cotonou, Benin; 6 Department of Public Health, Faculty of Medicine and Biomedical Sciences, University of Yaounde I, Yaounde, Cameroon; 7 Faculty of Health Sciences, Walter Sisulu University, Eastern Cape, South Africa; 8 Department of Basic Sciences, Unit of Clinical Pharmacology and Pharmacovigilance, Faculty of Medicine, University of Kinshasa, Kinshasa, DR Congo; 9 Department of Internal Medicine, Faculty of Medicine, University of Kinshasa, Kinshasa, DR Congo; 10 Department of General Practice, KU Leuven, Leuven, Belgium; 11 Department of Public Health, University of Kinshasa, Kinshasa, DR Congo; 12 Department of Public Health, University of Copenhagen, Copenhagen, Denmark; 13 Ministry of Health and Social Welfare, Monrovia, Liberia; 14 Department of Medicine, Usmanu Danfodiyo University Teaching Hospital, Sokoto, Nigeria; 15 Department of Medicine & Physiology, Faculty of Medical Sciences, University of Nigeria, Enugu Campus/University of Nigeria Teaching Hospital, Ituku Ozalla, Enugu, Nigeria; 16 Department of Medicine, College of Health Sciences, Bayero University Kano/Aminu Kano Teaching Hospital, Kano State, Kano, Nigeria; 17 Department of Medicine, College of Medical Sciences, University of Jos, Jos, Nigeria; 18 Department of Internal Medicine, University of Calabar/University of Calabar Teaching Hospital, Calabar, Nigeria; 19 Department of Medicine, Ekiti State University, Ado-Ekiti, Nigeria; 20 Department of Medicine, College of Medicine, University of Lagos, Lagos, Nigeria; 21 Division of Cardiovascular Medicine, Department of Medicine (University College Hospital), College of Medicine, University of Ibadan, Ibadan, Nigeria; 22 Institute of Social and Preventive Medicine, University of Lausanne, Lausanne, Switzerland; 23 University of the Free State, Bloemfontein, Republic of South Africa; 24 MRC Epidemiology Unit, University of Cambridge, Cambridge, UK; 25 Northumbria Healthcare NHS Foundation Trust, North Tyneside General Hospital, North Shields, Tyne and Wear, UK; 26 Institute of Health and Society, Newcastle University, Newcastle, UK; 27 National NCD Program, Ministry Of Health, Lome, Togo; 28 School of Medicine, The Copperbelt University, Ndola, Zambia; 29 Physiology Department, University of Zimbabwe, College of Health Sciences, Harare, Zimbabwe; 30 Department of Physiology, Muhimbili University of Health and Allied Sciences, Dar es Salaam, Tanzania; 31 HART (Hypertension in Africa Research Team), North-West University, Potchefstroom, South Africa; 32 Africa Unit for Transdisciplinary Health Research (AUTHeR), North-West University, Potchefstroom, South Africa; 33 MRC Unit for Hypertension and Cardiovascular Disease, North-West University, Potchefstroom, South Africa; 34 Department of Nutrition and Dietetics, University of the Free State, Bloemfontein, South Africa; 35 Wellcome Trust Africa Centre for Health and Population Studies, University of KwaZulu-Natal, KwaZulu-Natal, South Africa; 36 MRC/UVRI Uganda Research Unit on AIDS, Entebbe, Uganda; 37 MRC/Wits Developmental Pathways for Health Research Unit (DPHRU), University of the Witwatersrand, Johannesburg, South Africa; 38 Department of Chemical Pathology, National Health Laboratory Service, University of the Witwatersrand Medical School, Johannesburg, South Africa; 39 Department of Diabetes and Endocrinology, Nelson R. Mandela School of Medicine, University of KwaZulu-Natal, Durban, South Africa

## Abstract

**Background:**

Waist circumference (WC) thresholds derived from western populations continue to be used in sub-Saharan Africa (SSA) despite increasing evidence of ethnic variation in the association between adiposity and cardiometabolic disease and availability of data from African populations. We aimed to derive a SSA-specific optimal WC cut-point for identifying individuals at increased cardiometabolic risk.

**Methods:**

We used individual level cross-sectional data on 24 181 participants aged ⩾15 years from 17 studies conducted between 1990 and 2014 in eight countries in SSA. Receiver operating characteristic curves were used to derive optimal WC cut-points for detecting the presence of at least two components of metabolic syndrome (MS), excluding WC.

**Results:**

The optimal WC cut-point was 81.2 cm (95% CI 78.5–83.8 cm) and 81.0 cm (95% CI 79.2–82.8 cm) for men and women, respectively, with comparable accuracy in men and women. Sensitivity was higher in women (64%, 95% CI 63–65) than in men (53%, 95% CI 51–55), and increased with the prevalence of obesity. Having WC above the derived cut-point was associated with a twofold probability of having at least two components of MS (age-adjusted odds ratio 2.6, 95% CI 2.4–2.9, for men and 2.2, 95% CI 2.0–2.3, for women).

**Conclusion:**

The optimal WC cut-point for identifying men at increased cardiometabolic risk is lower (⩾81.2 cm) than current guidelines (⩾94.0 cm) recommend, and similar to that in women in SSA. Prospective studies are needed to confirm these cut-points based on cardiometabolic outcomes.

## Introduction

The rapidly increasing burden of cardiometabolic disease in sub-Saharan Africa (SSA) requires effective cardiometabolic disease prevention and management strategies.^1, 2, 3^ Detection of increased cardiometabolic risk in apparently healthy individuals is essential for timely intervention to help prevent or delay progression to disease.^4^ Given the resource constraints in SSA, there is a need for low cost and easily accessible tools for identifying individuals at increased risk to facilitate early initiation of lifestyle modification and/or treatment as part of cardiometabolic disease prevention and management programmes.

Anthropometric indices are cheap and simple tools used for assessment of overweight and obesity in clinical practice and have been shown to be good predictors of cardiovascular risk.^5^ In particular, raised waist circumference (WC), a marker of central obesity, is used independently or in conjunction with other risk factors to predict cardiometabolic disease.^6^ Compared with other anthropometric measures, WC is the cheapest, easiest to determine and, in some populations, the strongest anthropometric cardiometabolic risk predictor.^7^

The WC thresholds or cut-points derived from populations of European ancestry were recommended for assessing cardiometabolic risk in Africans in the absence of sufficient data from African populations.^8, 9^ These cut-points have continued to be used in SSA despite increasing availability of data from the region and growing evidence of ethnic variation in the relationship between adiposity and cardiometabolic risk.^6, 10^ Ethnic differences in the relationship between adiposity and cardiometabolic risk mean that the optimal WC threshold indicating increased cardiometabolic risk in SSA populations are likely to be different from those determined in western populations. Indeed, recent studies have indicated different WC thresholds in some SSA populations.^11, 12^ However, these studies have been characterised by relatively small sample size and highly homogeneous populations, mostly from South Africa. This has limited the adoption of their recommendations across SSA. We, therefore, aimed to derive an optimal WC threshold relevant for identifying individuals at increased cardiometabolic risk across the region using pooled individual participant data.

## Materials and methods

### Data sources and inclusion criteria

This study utilises data collated as part of the African Partnership for Chronic Disease Research (APCDR) (www.apcdr.org), an initiative that facilitates collaborative epidemiological and genomic research of chronic diseases across SSA. Specifically, this study draws on individual participant data collated from studies in SSA to assess the relationship between anthropometric variables and cardiometabolic disease risk. This is referred to subsequently in this paper as the ‘Anthropometry Study’. The following procedures were used to identify appropriate data sets to be included in the study. First, a literature search was conducted to identify published population-based studies that have collected data on anthropometric measurements and other cardiometabolic risk factors until December 2014. Second, all countries that had conducted STEPwise approach to chronic disease risk factor Surveillance surveys (STEPS) up to December 2014 were identified through a search of the literature and enquiry from the Department of Chronic Diseases and Health Promotion at the World Health Organisation (WHO) in Geneva. The lead investigators involved in these studies were contacted and invited to contribute to individual participant data for pooled analyses. Additional data sets were identified through communication with the initial investigators contacted. Supplementary Table S1 shows a summary of the data collated. Only individuals aged 15 years or older, not pregnant, and who had data on all five components of metabolic syndrome (MS) were included in our analysis. MS was defined according to the International Diabetes Federation (IDF) 2009 Joint Interim Statement (JIS) modified to allow for determination of glycaemic status using glycated haemoglobin (HbA1c) in the absence of fasting blood glucose.^6, 13, 14^

### Data collection

We used data collected on anthropometric measurements and other cardiometabolic risk factors in the Anthropometry Study. In the majority of the studies, anthropometric measurements were taken according to WHO guidelines using standardised and calibrated equipment.^15^ Blood samples for measurement of glucose and lipids were drawn after 8–10 h overnight fast except in one study that collected non-fasting samples for lipids and HbA1c.^16^ Details of measurements are shown in Supplementary Table S2.

### Definitions

We used the IDF harmonised criteria for MS to define cardiometabolic risk factors with a slight modification allowing for use of HbA1c to determine glycaemic status in the absence of fasting glucose.^6^ Raised WC was defined as WC ⩾94 cm (men) and WC ⩾80 cm (women); raised blood pressure (BP) as BP ⩾130/85 mm Hg or use of antihypertensive medication; low plasma high-density lipoprotein cholesterol (HDL-C) cholesterol as HDL-C <1.0 mmol l^−1^ in men and HDL-C <1.3 mmol l^−1^ in women. Raised plasma triglycerides (TG) was defined as TG>1.7 mmol l^−1^ and raised fasting blood/plasma glucose (FG) as FG⩾5.6 mmol l^−1^ or treatment for diabetes, or HbA1c⩾5.7% in the absence of fasting glucose.^6, 13, 14^ Raised body mass index (BMI) was defined as BMI ⩾25 kg m^−^^2^; obesity as BMI ⩾30 kg m^−^^2^; raised waist-to-hip ratio (WHR) as WHR>1.0 (men) and WHR>0.85 (women); raised waist-to-height ratio (WHtR) as WHtR>0.5; raised total plasma cholesterol (TC) as TC>5.0 mmol l^−1^; and raised plasma low-density lipoprotein cholesterol (LDL-C) as LDL-C>3.0 mmol l^−1^.^17, 18, 19^

### Statistical analysis

The full data set (obtained by merging data sets from all the contributing studies) for this study was split into two parts; one for derivation of optimal cut-points for anthropometric markers of adiposity, and one for validation of the derived cut-points. One study from each of the regions East Africa (Kenya, Tanzania and Uganda), West Africa (Nigeria and Benin) and Southern Africa (South Africa) was randomly selected for the validation data set while the remaining studies were used for derivation. This resulted in distribution of 18/82% of the full data set between the validation and derivation data sets, respectively.

For each of the data sets (full, derivation and validation), descriptive statistics, including means and prevalence of continuous and categorical variables, respectively, were calculated and presented with 95% confidence intervals (95% CI) for men and women separately, and for both sexes combined. Estimates of prevalence were adjusted to the WHO world population using the direct method to facilitate direct comparison between studies.^20^

Non-parametric receiver operating characteristics curve analyses were conducted using the derivation data set to assess the ability of anthropometric markers of adiposity (WC, BMI, WHR and WHtR) to detect the presence of at least two components of MS excluding WC, which aligns with previous studies.^12^ The area under the receiver operating characteristics curve (AUC) and the corresponding 95% CI were used to summarise the discriminatory power of each marker and the optimal cut-point was determined as the value corresponding to the Youden index. We used likelihood ratio tests to compare AUC of the other anthropometric markers with AUC for WC. The performance of the derived cut-points in terms of sensitivity, specificity, positive predictive value (PPV) and negative predictive value was then assessed in the validation data set and compared with thresholds currently recommended for adiposity in this population.

Sensitivity analyses were performed to assess whether the optimal WC cut-point depends on the prevalence of obesity; whether the exclusion of low HDL-C, because of its very high prevalence (age-adjusted 56%) affected the discriminatory power of WC; and whether the sensitivity of the derived cut-point for WC varied with age. Additionally, we assessed the relative probability of having at least two components of MS between individuals with a WC equal to or greater than the derived cut-point and individuals with a WC below the cut-point. We also compared WC cut-points determined by the Youden Index with WC cut-points based on inflexion points at which the odds ratio of having at least two components of MS suddenly increases. Furthermore, we conducted sensitivity analyses to assess whether the inclusion of adolescents (15–18 years) had an impact on derived cut-points for WC by comparing cut-points derived with and without this age group.

All analyses were performed in STATA 13.1 (Stata, College Station, TX, USA).

## Results

**Figure 1:**
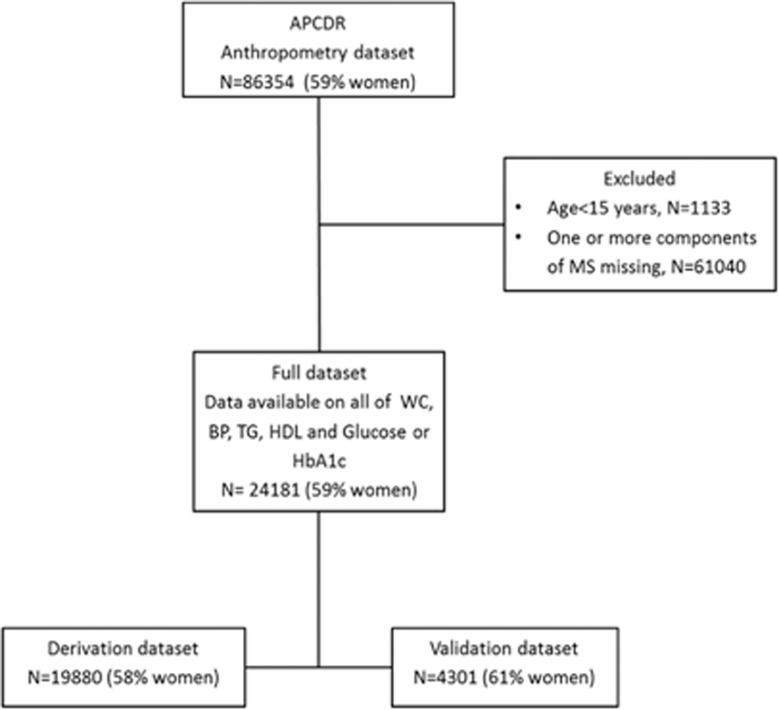
Data sets used for derivation and validation of cut-points of adiposity markers to define metabolic syndrome.

The APCDR Anthropometry study data set comprises 41 studies with a total of 86354 (59% women) (Figure 1) individuals aged 1–115 years. Of these, we included in our analyses 24181 (59% women) from 17 studies in eight countries (Benin, Nigeria, Democratic Republic of Congo, Uganda, Kenya, Tanzania, South Africa and Seychelles) who were ⩾15 years of age and had data on all of WC, BP, plasma TG, plasma HDL-C and fasting blood/plasma glucose or HbA1c. Table 1 summarises the characteristics of individuals in the current study. The overall mean age was 41.9 years (41.7–42.1) with only a slight difference between women and men (women: 42.2 years (42.0–42.5) versus men: 41.5 years (41.2–41.8)). Compared with men, women also had a significantly higher mean BMI, WC, hip circumference, WHtR, diastolic BP, TC, LDL-C, FG and HbA1c, while there was no sex difference in mean TG and HDL-C. The mean WC was 79.0 cm (range 49–180 cm) among men, and 80.9 cm (range 53–171 cm) in women. Conversely, men had a higher mean WHR and systolic BP. Further, there was no sex difference in age-adjusted prevalence of raised TG, while the prevalence of low HDL-C was higher in women than in men. The age-adjusted prevalence of MS was 20% (20–21%) with a significantly higher prevalence in women (26% (26–27%)) than in men (11% (11–12%)), and in older age groups than younger age groups (Supplementary Figure S1).

Similarly, the prevalence of individual cardiometabolic risk factors was higher in women and older individuals (Supplementary Figure S2). The age-adjusted prevalence of abdominal obesity determined by raised WC was 35% (34–35%) overall, but 50% (49–50%) in women and only 12% (11–12%) in men. The age-adjusted prevalence of total body obesity based on BMI (⩾30 kg m^−^^2^) was 15% (14–15%) overall, 6% (6–7%) in men and 23% (22–23%) in women. The age-adjusted prevalence of raised blood pressure was 40% (40–41%) overall and only slightly higher in men than in women. Low HDL cholesterol was the most common type of dyslipidaemia with an overall age-adjusted prevalence of 57% (56–57%), 40% (39–41%) in men and 68% (67–68%) in women. Raised TG was the least common with an age-adjusted prevalence of 13% (13–13%) with no significant sex differences. The age-adjusted prevalence of raised blood glucose was 17% (16–17%) and was also not significantly different between men and women (Table 1).

Table 2 gives the results from the derivation data set of receiver operating characteristics curve analyses for identifying the optimal cut-points of selected adiposity measures for detecting the presence of at least two components of MS (excluding WC). The optimal cut-point for WC was 81.2 cm (78.5–83.8 cm) in men; this was not statistically significantly different from 81.0 cm (79.2–82.8 cm) derived for women. The corresponding AUCs were similar between men and women, 0.66 (0.65–0.68) in men and 0.66 (0.65–0.67) in women. Compared with each of the other anthropometric markers of adiposity (BMI, WHR and WHtR), WC had a greater or equal accuracy of predicting individuals with at least two MS components (Supplementary Figure S3). The sensitivity of the derived WC cut-point within the derivation data set was low but greater in women 64% (63–65%) than in men 53% (51–55%).

Table 3 shows the performance of the derived cut-points of WC and other anthropometric markers in the validation data set. The derived cut-point for WC in men (⩾81.2 cm) had a sensitivity of 60% (54–65%), which was higher than the sensitivity in the derivation data set 53% (51–55%). However, this sensitivity was higher than the sensitivity of 31% (26–36%) of the current cut-point (⩾94.0 cm). Generally, among men, derived cut-points showed higher sensitivities than currently recommended cut-points for all anthropometric indices of adiposity. Among women, the derived WC cut-point (⩾81.0 cm) had a sensitivity of 67% (64–70%). This was higher than the sensitivity in the derivation data set (64%, (63–65%)), but slightly lower than the sensitivity of the currently recommended threshold (⩾80.0 cm, sensitivity: 71% (68–74%)). The derived cut-point for WHtR (>0.54) had a significantly lower sensitivity in the validation data set (55% (52–58%)) than the currently recommended cut-point (>0.50) for women. However, the sensitivity (66% (62–69%)) of the derived cut-point for WHR (>0.83) was higher than the sensitivity (54% (50–57%)) of the currently recommended cut-point (>0.85) (Table 3). The PPV associated with the derived WC cut-points were 46% (44–49%) for women and 35% (32–39%) for men. The negative predictive value was high in both men 86% (84–88%) and women 77% (74–79%). As expected, the PPV increased with the prevalence of at least two components of MS across studies. Among women, the PPV ranged from 21 to 80% when the prevalence of the presence of at least two components of MS was 15 and 72%, respectively (Supplementary Table S3). In men, the PPV ranged from 19 to 76% when the prevalence of the presence of at least two components of MS was 10% and 48%, respectively.

### Sensitivity analyses

**Figure 2:**
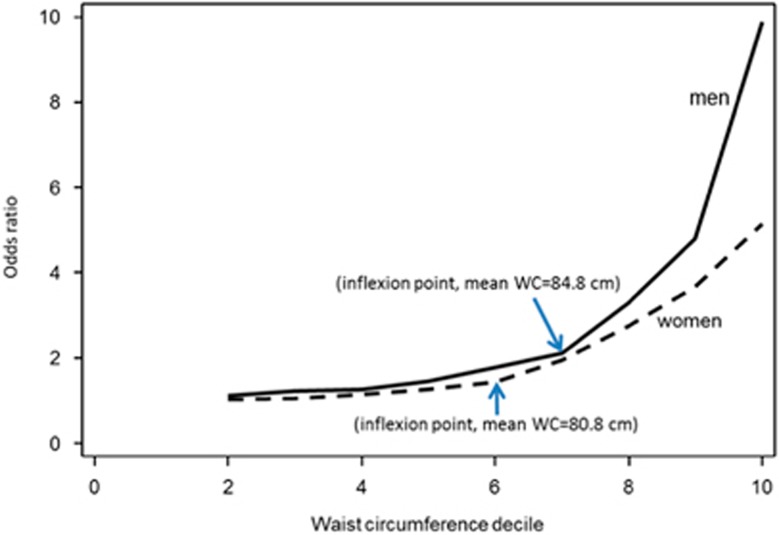
Odds ratio of having at least two components of metabolic syndrome (MS) in each decile (second to tenth) of waist circumference relative to the first decile (number of participants, 19 880: men 8055, women 11 825).

Results of sensitivity analyses are shown in Supplementary Figures S4–S9. They show a positive correlation between prevalence of obesity and the optimal WC cut-point (Supplementary Figures S4 and S5); a higher AUC when low HDL-C is excluded as a component of MS (Supplementary Figure S6), as well as age-peaks for the sensitivity of the derived optimal WC cut-point in men and women (Supplementary Figure S7). Additionally, individuals with WC greater than or equal to the derived cut-point were two times more likely to have at least two components of MS (adjusting for age) (men, OR 2.6, 95% CI 2.4–2.9; women, OR 2.2, 95% CI 2.0–2.3), compared with individuals with WC below the cut-point (Supplementary Figure S8). Further, the inflexion point-based WC cut-point (Figure 2) was similar to the cut-point based on Youden Index for women (80.8 cm compared with 81.0 cm 95% CI 79.2–82.8, respectively) but different for men (84.8 cm compared with 81.2 cm 95% CI 78.5–83.8, respectively). However the sensitivity of the cut-point of 84.8 cm was only 38%, much lower than 58% for the cut-point of 81.2 cm, among men. In addition, the derived optimal WC cut-point excluding adolescents aged 15–18 years (6.9% of the derivation data set) was 81.6 cm (95% CI 78.8–84.4 cm) for men and 81.2 cm (95% CI 78.9–83.5 cm), which were not significantly different from the cut-points derived from data including adolescents.

## Discussion

In this pooled analysis of 24181 participants, we have derived WC cut-points relevant for identifying men and women at increased cardiometabolic risk in populations across SSA. The derived optimal WC cut-point for women is similar to the currently recommended threshold for Africans (81.0 versus 80.0 cm) but substantially lower for men (81.2 versus 94.0 cm). The findings suggest that current WC guidelines underestimate cardiometabolic risk among African men. Importantly, as a consequence, current guidelines may underestimate the burden of abdominal obesity by as much as 7 percentage points in the general population and 22 percentage points among men in SSA. This could have wider implications including inadequate resource allocation for prevention and control of obesity and the initiation of lifestyle interventions too late in the disease process. This emphasises the need to cautiously interpret estimates of disease risk and burden based on indicators derived for a different population. Therefore, the findings of this study provide evidence of the need to undertake prospective studies to establish more broadly the relationship between cardiometabolic risk factors and clinical events in SSA.

The markedly lower cut-point for WC (and other markers of adiposity including BMI, WHR and WHtR) in men highlights ethnic differences in the relationship between anthropometry, adiposity and cardiometabolic risk. This cut-point is probably a reflection of a stronger association between WC (a proxy for visceral adiposity) and cardiometabolic risk among African men compared with men of European descent from whom the cut-point that was recommended for SSA was derived.^21, 22^ Similar observations in Asian populations have been explained by greater visceral adiposity in Asians compared with Europeans at the same level of WC.^23, 24, 25^ This explanation is unlikely to hold in the case of African men as available evidence suggests that populations of African ancestry (African Americans and Afro-Caribbeans) have less visceral fat compared with populations of European ancestry at a given level of WC.^26, 27^ Therefore, other aetiological factors, such as the impact of exposure to undernutrition (including gestational exposure to maternal undernutrition) on subsequent weight gain, adiposity and adipocyte secretion profiles, perhaps interacting with genetic predisposition, may explain a stronger association between WC and metabolic risk in African populations.^8^ Furthermore, we found low HDL-C to be the most common cardiometabolic risk factor and raised TG the least common. This is in contrast to evidence from European populations, where cardiometabolic risk is characterised by hypertriglyceridaemia, while low TG is a consistent characteristics of people of African descent in the same environment.^28, 29^ Thus, our finding may reflect this ethnic variation in cardiometabolic risk profiles and their relationship with measures of adiposity.

Although previously indicated in smaller, homogeneous African populations, our study is the largest and the most population-diverse to demonstrate a lower WC cut-point for identifying men at increased cardiometabolic risk in SSA compared with men of European descent.^12^ Notably, the cut-point derived in our study is lower than that reported in earlier studies. A possible explanation, given that WC is not only a reflection of subcutaneous and visceral fat accumulation in the trunk region but also of absolute body size, would be that our study included younger participants (adolescents aged 15–18 years) who more likely to have a smaller absolute body size compared with adults, while the minimum age in earlier studies was 25 years. However, sensitivity analyses in our study found no significant impact of including adolescents aged 15 years or older. Further, as discussed, the majority of the earlier reports were from South Africa, where the underlying prevalence of obesity is much higher relative to our study. The current study includes South African cohorts and populations with a low prevalence of obesity from outside of South Africa. Importantly, greater variation in urbanisation and lifestyles due to differences in stages of socio-economic transition in the present study may also explain some of the observed differences. However, the optimal WC cut-point for detecting the presence of at least two components of MS and the optimal cut-point for individual risk factors among men were broadly similar in the current study with the exception of raised FG/HbA1c and low HDL which had higher individual cut-points.

Among women, our findings suggest that the cut-point recommended in current guidelines may be appropriate in this population. This is consistent with the findings of a previous study which found no evidence of racial differences in the association between BMI and WC among women.^30^ The implication of this is that current WC guidelines, which are based on BMI among women of European descent, may be appropriate for women in SSA. However, we note that studies in populations with a high prevalence of obesity have reported markedly higher WC cut-points for men and most particularly women.^11, 12, 31^ The explanation for this is not clear, but these data do suggest that in populations with high levels of obesity, the cardiometabolic components of MS become prevalent at a higher level of abdominal obesity than observed in populations with a lower prevalence of obesity. Additionally, the optimal WC cut-point for individual risk factors except, as in men, low HDL and raised FG/HbA1c, was, in contrast with the observation among men, generally higher than the optimal WC for detecting the presence of at least two components of MS among women.

Further, our findings suggest greater utility of WC compared with other markers of adiposity (BMI, WHR and WHtR) in detecting the presence of at least two other components of MS. This is consistent with the growing body of evidence showing that WC, compared with other anthropometric measures, is a stronger indicator of visceral adiposity.^12, 32^ In the present study we also found WC to have the strongest association with the presence of at least two other cardiometabolic risk factors (raised blood pressure, components of dyslipidaemia and raised glycaemic levels) compared with BMI, WHR and WHtR in men and women (stronger in men than in women). This finding contrasts with other studies that have found WHR and WHtR to be more strongly associated with cardiovascular risk than WC.^33, 34^ These differences require further evaluation but they highlight the complexity of the relationship between anthropometric measures, adiposity and cardiometabolic disease risk. The pathogenesis of cardiometabolic disease likely involves other environmental and genetic risk factors whose effects may not be mediated through some measures of anthropometry.

Another notable finding from this study is the suggestion of a common WC cut-point for men and women. Whether this is an indication of comparable visceral adiposity and cardiometabolic risk at similar levels of WC in men and women in this population is not clear. Our data show that men, despite having a four times lower prevalence of obesity (BMI⩾30 kg m^−^^2^), have only a slightly higher prevalence of hypertension relative to women. This phenomenon has been observed elsewhere and is thought to be the confounding effect of smoking and alcohol consumption in men.^34, 35^ Regardless of the underlying mechanism, a common WC cut-point for men and women may have important implications for clinical practice and health promotion because it is easier to formulate a single health message for men and women. To our knowledge, only one other study has previously reported a common WC cut-point for men and women.^36^ Further assessment of this result in a prospective study in a population of similar diversity as the present study would help clarify this finding.

Our findings also suggest that the optimal WC cut-point and the associated sensitivity for identifying individuals at increased cardiometabolic risk may be influenced by age and BMI. Previous studies have similarly noted the positive effects of population mean BMI and WC levels on the optimal waist cut-point for MS diagnosis.^37, 38, 39^ Furthermore, given the low sensitivity and specificity of WC observed in this and previous studies, there may be need to consider the feasibility of age- and BMI-specific cut-points for identifying individuals at increased cardiometabolic risk.^40^

The strength of this study is its large sample size and geographical coverage. With over 24 000 participants drawn from Central, East, South and West Africa, this study is the largest and most diverse in terms of populations in SSA, to assess an optimal cut-point for WC to date. In addition, we validated the derived cut-points in a separate data set, and the comparable sensitivities and specificities observed suggest that the thresholds are reliable and valid. Further, two methods of determining the optimal cut-point were used and yielded broadly similar results which increases the reliability of cut-points derived in this study.

The study has some limitations. First, like cut-points currently in use, the cut-points derived in this study are based on cross-sectional data, which precludes examination of the temporality of the association between raised WC and cardiometabolic risk factors. This is important because it is possible that the development of diabetes or hypertension influences body size. Second, our analyses have been limited to examining the association of anthropometric markers of adiposity with cardiometabolic risk factors rather than cardiovascular events, diabetes or mortality. However, even the current WC cut-points (derived among populations of European ancestry) are based on their ability to detect overweight and obesity as defined by BMI and not their relationship with cardiometabolic risk.^41^ Additionally, the WC cut-points for individual risk factors (raised TG, HDL-C and fasting blood glucose) found in our study are similar to those previously reported in a prospective study of black South African women indicating that the cut-points we have derived may be acceptable approximate indicators of future cardiometabolic risk.^34^ Third, the HIV and antiretroviral therapy may have influenced our results. However such influences, if any, are likely to be limited to South Africa where the prevalence of HIV and antiretroviral therapy use is substantial. Further we used HbA1c to assess dysglycaemia; however, its use is yet to be validated in populations in SSA where factors that alter red cell lifecycle such as sickle cell disease and malaria are prevalent. We also recognise that variation in measurement of WC between studies due to differences in equipment and skill of people taking measurements, among others, might have influenced the validity of cut-off points derived in this study. However, the direction of this bias is unclear.

In summary, our findings indicate that men in SSA are likely to be at increased cardiometabolic risk at a lower WC threshold than recommended in current guidelines, while the threshold that was recommended for women may be appropriate. Thus, current guidelines could be substantially underestimating abdominal obesity in men in Africa, which has policy, public health and health-care implications. This reiterates the importance of population-specific anthropometric cut-points that account for ethnic variation in adiposity and its association with cardiometabolic risk.^38^ However, there is a need for prospective studies to clarify the impact of underlying population distribution of obesity on the optimal WC cut-point and to confirm these cut-points based on prospective risk of hard cardiometabolic outcomes. Future efforts in this respect should leverage existing cohorts and health surveillance systems in SSA.

## Figures and Tables

**Table 1:** Participant characteristics in the full data set (*N* 24 181: men 9729, women 14 452)

*Characteristics*^a^	N^b^ *(men/women)*		*Men*	*Women*	*All*
*Mean (95% CI)*					
Age (years)*	9729/14 452		41.5 (41.1–41.8)	42.2 (42.0–42.5)	41.9 (41.7–42.1)
WC**	9729/14 452		79.0 (78.8–79.2)	82.1 (81.9–82.3)	80.9 (80.7–81.0)
BMI**	9709/14 426		22.0 (21.9–22.1)	25.2 (25.1–25.3)	24.0 (23.9–24.0)
Hip*	9234/13 933		88.8 (88.6–89.1)	97.5 (97.1–97.8)	94.1 (93.9–94.3)
WHR**	9234/13 933		0.89 (0.89–0.89)	0.85 (0.85–0.85)	0.87 (0.87–0.87)
WHtR**	8919/13 230		0.48 (0.47–0.48)	0.53 (0.52–0.53)	0.51 (0.50–0.51)
SBP**	9535/14 273		125 (124–125)	123 (123–124)	124 (124–124)
DBP**	9703/14 432		77 (77–77)	78 (78–78)	78 (77–78)
TC**	7896/11 422		4.0 (4.0–4.0)	4.2 (4.2–4.2)	4.1 (4.1–4.1)
TG^c^	9729/14 452		1.00 (0.99–1.02)	0.99 (0.98–1.00)	1.0 (0.99–1.00)
HDL-C	9729/14 452		1.2 (1.2–1.2)	1.2 (1.2–1.2)	1.2 (1.2–1.2)
LDL-C**	6688/10 485		2.2 (2.2–2.2)	2.4 (2.4–2.4)	2.3 (2.3–2.4)
FG**	6693/10 571		5.1 (5.1–5.1)	5.2 (5.2–5.3)	5.2 (5.1–5.2)
HbA1c**	4073/5851		5.3 (5.2–5.3)	5.4 (5.4–5.4)	5.3 (5.3–5.4)
					
*Prevalence % (95% CI)*					
MS (⩾3 of 5 abnormalities)**	9729/14 452		11 (11–12)	26 (26–27)	20 (20–21)
WC⩾94/80 (men/women)**	9729/14 452		12 (11–12)	50 (49–50)	35 (34–35)
BMI⩾25**	9709/14 426		19 (19–20)	41 (41–42)	33 (32–33)
BMI⩾30**	9709/14 426		6 (5–6)	20 (20–21)	15 (14–15)
WHR>1.0/0.85 (men/women)**	9234/13 933		10 (9–11)	45 (44–46)	31 (30–32)
WHtR>0.5**	8919/13 230		27 (26–28)	54 (53–55)	43 (43–44)
BP ⩾130/85 or use of antihypertensive medication*	9729/14 452		42 (41–43)	39 (38–40)	40 (40–41)
TC>5.0**	7896/11 422		21 (20–21)	24 (23–24)	22 (22–23)
TG>1.7	9729/14 452		13 (12–14)	13 (13–14)	13 (13–13)
HDL-C <1.0/1.3 (men/women)**	9729/14 452		40 (39–41)	68 (67–68)	57 (56–57)
LDL-C>3.0**	6688/10 485		19 (18–20)	24 (23–24)	22 (21–22)
FG>5.6 or HbA1c ⩾5.7^d^	9729/14 452		17 (17–18)	16 (16–17)	17 (16–17)
Ever smoked**	6798/9400		24 (23–26)	5 (5–6)	13 (13–14)
Ever consumed alcohol**	4423/6367		51 (49–52)	35 (34–37)	41 (40–42)

Abbreviations: BMI, body mass index (kg m^−^^2^); BP, blood pressure (mmHg); CI, confidence interval; DBP, diastolic blood pressure (mmHg); FG, fasting blood/plasma glucose (mmol l^−1^); HbA1c, glycated haemoglobin (%); HDL-C, high-density lipoprotein cholesterol (mmol l^−1^); Hip, hip circumference (cm); LDL-C, low-density lipoprotein cholesterol (mmol l^−1^); *N*, number of participants; SBP, systolic blood pressure (mmHg); TC, total cholesterol (mmol l^−1^); TG, triglycerides (mmol l^−1^); WC, waist circumference (cm); WHR, waist-to-hip ratio; WHtR, waist-to-height ratio.

a Means and prevalence are standardised to the WHO world standard population using the direct method.

b The total of men and women for some characteristics is less than 24 181 because of missing data.

c Data are median standardised to the median age in the full data set.

d Individuals with both FG and HbA1c measurements available were classified using FG. **P<0.001, *P<0.05 (comparisons are between men and women). Data are mean (95% CI) (except as indicated by ^b^) and prevalence (%) (95% CI) (some CI limits coincide due to rounding errors).

**Table 2:** Results of the receiver operating characteristics (ROC) curve analyses for identifying optimal anthropometric cut-points for detecting the presence of at least two components of MS (excluding WC) in the derivation data set (*N* 19 880: men 8055, women 11 825)

*Anthropometric variable*	*AUC*	P-*value*^a^ *for difference in AUC between index and WC*	*Cut-point*	*Sensitivity (%)*	*Specificity (%)*	*Youden index*
*Overall*						
WC	0.66 (0.65–0.67)	—	81.1 (80.4–81.6)	61 (60–62)	65 (64–66)	0.260
BMI	0.63 (0.62–0.64)	<0.001	24.8 (23.9–25.6)	51 (50–52)	70 (70–71)	0.214
WHR	0.56 (0.55–0.57)	<0.001	0.87 (0.85–0.89)	51 (50–53)	59 (58–60)	0.103
WHtR	0.65 (0.64–0.66)	0.277	0.51 (0.49–0.53)	53 (52–55)	70 (69–71)	0.234
						
*Men*						
WC	0.66 (0.65–0.68)	—	81.2 (78.5–83.8)	53 (51–55)	73 (72–74)	0.258
BMI	0.64 (0.62–0.65)	<0.001	23.2 (22.1–24.3)	48 (46–50)	74 (73–76)	(0.220
WHR	0.59 (0.58–0.60)	<0.001	0.88 (0.85–0.90)	62 (60–64)	53 (51–54)	0.156
WHtR	0.65 (0.64–0.67)	0.276	0.48 (0.47–0.49)	58 (56–60)	66 (65–68)	0.242
						
*Women*						
WC	0.66 (0.65–0.67)	—	81.0 (79.2–82.8)	64 (63–65)	61 (60–62)	0.251
BMI	0.62 (0.61–0.63)	<0.001	25.1 (23.6–26.6)	57 (55–58)	65 (64–66)	0.193
WHR	0.56 (0.55–0.57)	<0.001	0.83 (0.81–0.85)	62 (60–63)	49 (48–51)	0.114
WHtR	0.65 (0.64–0.66)	<0.001	0.54 (0.53–0.56)	54 (53–56)	69 (68–70)	0.229

Abbreviations: AUC, area under the curve; BMI, body mass index (kg m^−^^2^); *N,* number of participants; WC, waist circumference (cm); WHR, waist hip ratio; WHtR, waist-to-height ratio.

a *P*-values reported for tests restricted to individuals in which both WC and the anthropometric measure assessed are determined. Data in brackets are 95% confidence intervals.

**Table 3:** Performance of derived cut-points compared with current cut-points in the validation data set (*N* 4301: men 1674, women 2627)

*Anthropometric variable*	*Derived/current index*	N^a^	*Cut-point*	*Sensitivity (%)*	*Specificity (%)*	*Positive predictive value (%)*	*Negative predictive value (%)*
*Men*							
WC	Derived	1674	81.2	60 (54–65)	69 (64–72)	35 (32–39)	86 (84–88)
	Current	1674	94	31 (26–36)	93 (92–94)	56 (49–63)	83 (81–85)
BMI	Derived	1674	23.2	41 (35–46)	79 (77–81)	35 (31–40)	82 (80–85)
	Current	1674	25	31 (26–36)	88 (86–89)	41 (35–47)	82 (80–84)
WHR	Derived	1373	0.88	65 (60–70)	56 (53–59)	33 (29–36)	84 (81–86)
	Current	1373	1	12 (9–16)	98 (97–99)	70 (56–81)	78 (75–80)
WHtR	Derived	1671	0.48	57 (52–62)	68 (66–71)	34 (30–38)	85 (83–87)
	Current	1671	0.5	50 (44–55)	78 (76–80)	39 (34–43)	85 (82–87)
							
*Women*							
WC	Derived	2627	81	67 (64–70)	58 (56–60)	46.3 (44–49)	77 (74–79)
	Current	2627	80	71 (68–74)	54 (52–57)	45 (43–48)	78 (75–80)
BMI	Derived	2624	25.1	57 (53–60)	71 (69–73)	51 (48–54)	75 (73–77)
	Current	2624	25	59 (56–63)	69 (67–72)	51 (48–54)	76 (74–78)
WHR	Derived	2289	0.83	66 (63–69)	47 (45–50)	42 (39–44)	71 (68–74)
	Current	2289	0.85	54 (50–57)	61 (58–63)	44 (41–47)	70 (67–72)
WHtR	Derived	2626	0.54	55 (52–58)	73 (71–75)	53 (49–56)	75 (73–77)
	Current	2626	0.5	71 (68–74)	54 (52–57)	45 (43–48)	78 (75–80)

Abbreviations: BMI, body mass index (kg m^−^^2^); *N*, number of participants; WC, waist circumference (cm); WHR, waist hip ratio; WHtR, waist-to-height ratio.

a The total of men and women for some indices is less than 4301 because of missing data. Data in brackets are 95% confidence intervals.
