# Supplementary material for: Deriving an optimal threshold of waist circumference for detecting cardiometabolic risk in sub-Saharan Africa
Source: Int J Obes (Lond). 2017 Oct 31;42(3):487–94. doi: 10.1038/ijo.2017.240 (PMC5880575; doi:10.1038/ijo.2017.240)
Supplement: Supplementary Table 1 [file ijo2017240x1.docx]

| **Table S1. Summary of the African Partnership for Chronic Disease Research Anthropometry dataset.** | | | | | | | | | |
| --- | --- | --- | --- | --- | --- | --- | --- | --- | --- |
|  | **Contributor** | **Country** | **Study** | **Setting and Population** | **Year** | **Partici-pants** | **Anthrop-ometry Measure** | **CMD variables** | **Notes** |
| 1 | Delisle-Sossa^1^ | Benin | Population based survey‡ | Cotonou (urban), Ouidah (small urban) and Ouidah rural, men & women aged 25-60 years | 2006 | 541 | WC, BMI | BP, glucose, TC, TG, HDL-C, LDL-C, Insulin | Fasting samples |
| 2 | Assah^2^ | Cameroon | Populaion based survey (Cameroon Burden of Diabetes (CamBoD) Baseline Survey) | National survey; men & women aged 15-99 years | 2003 | 10011 | WC, BMI, WHR | BP, OGTT, FBG | Fasting samples |
| 3 | On’Kin^3^ | DR Congo | Population based survey | Kinshasa (urban), men & women aged 13-98 years | 2005 | 973 | WC, BMI | BP, glucose | Fasting samples |
| 4 | Nzambi | DR Congo | Population based survey‡ | Gombe Matadi (rural) Bas Congo Province, men & women aged 7-88 years | 2011 | 1500 | WC, BMI | BP, glucose | Fasting samples |
| 5 | Longo-Mbenza^4^ | DR Congo | Population based survey (STEPS)‡ | Kinshasa; men & women aged 15-86 | 2005 | 2000 | WC, BMI | BP, glucose | Fasting glucose |
| 6 | Buntix^5^ | DR Congo | Population based survey on diabetes prevalence | Kisantu; men & women aged 20-97 years | Not provided | 1759 | WC, height | glucose, OGTT, BP | Multi-stage random sample; Fasting samples |
| 7 | Christensen^6^ | Kenya | Population based study on glucose intolerance‡ | Bondo, Kitui, Transmara and Nairobi; men & women aged 17-68 years | 2005-06 | 1438 | WC, BMI, WHR | BP, glucose, OGTT, HbA1c, HDL-C, LDL-C, TG, TC, VLDL | Fasting samples |
| 8 | Wesseh^7^ | Liberia | Population based survey (STEPS) | 5 National counties; men & women aged 24-64 years | 2011 | 2671 | WC, BMI, WHR | BP | Multi stage sampling, randomly selected 5 counties |
| 9 | Sabir^8^ | Northern Nigeria | Population based survey | Northern Nigeria, Hausa Fulani population; men & women aged 16-65 years | Not provided | 825 | WC, BMI | BP | Multi stage sample |
| 10 | Okafor^9^ | Nigeria | Population based survey | Enugu (urban), Southeast Nigeria; Igbo population, men & women aged 18-70 years | 2006 | 898 | WC, BMI, WHR | BP | Multi stage sample |
| 11 | Gezawa^10^ | Nigeria | Population based study | Maiduguri, Northeastern Nigeria (urban); men & women aged 15-70 years | 2008 | 1650 | WC, BMI, WHR | BP | Multi stage sample |
| 12 | Puepet | Nigeria | Population based study | Jos sub-urban; men & women aged 20-89 years | 2006 | 709 | WC, BMI | glucose | Fasting samples |
| 13 | Puepet | Nigeria | Population based study | Jos urban; men & women aged 10-85 years | 2012 | 753 | WC, BMI | BP, glucose | Fasting samples |
| 14 | Puepet | Nigeria | Population based study | North central Nigeria; men & women aged 30-82 years | 2009 | 646 | WC, BMI | TC, HDL-C, TG, HbA1c, albumin, creatinine | Fasting samples |
| 15 | Ofem-Enang^11^ | Nigeria | Population based study | Calabar city (urban), Cross River state Nigeria; men & women aged 16-73 | 2011 | 1134 | WC, BMI | BP, glucose | Fasting samples |
| 16 | Taiwo | Nigeria | Population based study | Location not provided; men & women aged 18-70 | Not provided | 929 | WC, BMI, WHR | BP |  |
| 17 | Ohwovoriole | Nigeria | Population based study | Calabar in South Nigeria, (rural/urban); men & women aged 16-31 years | Not provided | 604 | WC, BMI | BP, glucose | Fasting samples |
| 18 | Oladapo | Nigeria | Population based study‡ | Ibadan (urban); men & women aged 20-100 | 2006 | 1220 | WC, BMI, WHR | BP, glucose, TC, HDL-C, LDL-C, TG | Fasting samples |
| 19 | Bovet^12-15^ | Seychelles | Population based surveys‡ | Independent sex- and age-stratified random samples of population aged 25-64 from Mahé (=90% of total population) in 1989, 1994, 2004) and from all islands (2013); (rural and urban) | 1989,1994, 2004, 2013 | 4632 | WC, BMI, WHR | BP, glucose, HDL-C, LDL-C, TG, TC | (1989[1081],1994[1067], 2004[1255], 2013[1240]) Fasting samples |
| 20 | Motala^16^ | South Africa | Population based study‡ | Ubombo district of the province of KwaZulu-Natal (rural), African (black) community of Zulu descent; men & women aged ≥15 years | 1999 | 1025 | WC, BMI, WHR | BP, glucose, TC, TG, HDL-C, LDL-C | Fasting samples |
| 21 | Crowther^17^ | South Africa | Survey of participants in the Birth to Twenty cohort study‡ | Soweto, Johannesburg (urban); African women aged 18-84 years | 2003 | 1251 | WC, BMI, WHR | Glucose, Insulin, HDL-C, LDL-C, TC, TG, BP | Fasting samples |
| 22 | Mollentze^18^ | South Africa | Population based study‡ | Black population in rural (QwaQWa), urban (Mangaung) Orange Free State; men & women aged 24-103 years | 1990 | 1611 | WC, BMI, WHR | TC, HDL-C, LDL-C, TG, glucose, BP | Fasting samples |
| 23 | Thorogood^19^ | South Africa | Population based survey | SASPI, Agincourt (rural); men & women aged 35-95 years | Not provided | 402 | WC, BMI | cholesterol, HDL-C, glucose, BP | Fasting samples |
| 24 | Walsh^20^ | South Africa | Population based survey‡ | AHA in the Free State (AHA-FS study) (rural); men & women aged 25-64 years | 2009 | 697 | WC, BMI | BP, glucose, HDL-C, LDL-C, TG, TC | Cross-sectional baseline survey for AHA; Fasting samples |
| 25 | Walsh^20^ | South Africa | Population based survey‡ | AHA in the Free State (AHA-FS study) (urban); men & women aged 25-64 years | 2009 | 565 | WC, BMI | BP, glucose, HDL-C, LDL-C, TG, TC | Cross-sectional baseline survey for AHA; Fasting samples |
| 26 | Pillay^21^ | South Africa | Population based survey | Health and demographic surveillance system of ACHPS in Durban (rural ); men & women resident in the HDSS area, aged 9-73 | 2003 | 4058 | BMI | BP |  |
| 27 | Pillay^21^ | South Africa | Population based survey | Health and demographic surveillance system of ACHPS in Durban (rural ); men & women resident in the HDSS area, aged 12-115 | 2010 | 13415 | BMI | BP |  |
| 28 | Motala/Sandhu^22^ | South Africa | Population based survey‡ | Durban Diabetes Study, e Thekwini Municipality, Durban, KwaZulu-Natal; men & women of African descent aged ≥18 years | 2014 | 1204 | WC, BMI, WHR | BP, FBG, HDL-C, LDL-C, TG, HbA1c | Fasting samples |
| 29 | Schutte^23^ | South Africa | Population based study‡ | PURE study, North-west province; men & women aged 35-70 years | 2005 | 2028 | WC, BMI, WHR | BP, glucose, HbA1c, TC, HDL-C, LDL-C, TG | Fasting samples |
| 30 | Kruger^24^ | South Africa | Population based study‡ | THUSA study, North West province; Black people; men & women aged 15-90 | 2004 | 1820 | WC, BMI, WHR | TC, TG, HD-CL, LDL-C, glucose, BP | Fasting samples |
| 31 | Njelekela^25^ | Tanzania | Population based study‡ | Residents of Dar es Salaam (urban); men & women aged 45-66 years | 2006 | 209 | WC, BMI, WHR | BP, glucose, TC,TG, HDL-C | Fasting samples |
| 32 | Unwin | Tanzania | Population based study‡ | Hai (rural, near Kilimanjaro) and Dar es Salaam (urban); men & women aged 15-102 | 1996/7 | 1644 | WC, BMI, WHR | BP, glucose | Fasting samples |
| 33 | Unwin | Tanzania | Population based study | Migration study, Morogoro (rural); men & women aged 15-59 | 2003 | 418 | WC, BMI, WHR | BP, glucose, TC, TG, HDL-C | Baseline data from the Urbanization and Metabolic Outcomes Study in Tanzania; Fasting samples |
| 34 | Walker/Gray^26^ | Tanzania | Population based study | Stroke incidence study, Hia (rural) and Daressalaam (Urban); men & women aged 15-112 |  | 598 | BMI | BP, glucose, TC, TG, HDL-C | Non-fasting samples |
| 35 | Walker/Gray^27^ | Tanzania | Population based study | Hai (rural); men & women aged ≥ 70 years |  | 2232 | BMI | BP |  |
| 36 | Agoudavi^28^ | Togo | Population based survey (STEPS) | Location not provided; men & women aged 15-65 years | 2010 | 4650 | WC, BMI | BP, glucose, TC | Fasting samples |
| 37 | Kaleebu/Sandhu^29^ | Uganda | Population based survey‡ | General Population Cohort, south-Western Uganda (rural); men & women aged ≥13 years | 2010 | 7552 | WC, BMI, WHR | BP, TC, TG, HDL-C, LDL-C, HbA1c | Survey conducted in a general population cohort (GPC); Non-fasting lipids |
| 38 | Siziya^30^ | Zambia | Population based survey (STEPS) | Kaoma and Kasama (rural); men & women aged 25-99 | 2008, 2009 | 2093 | WC, BMI | BP, glucose, TC, TG | Multistage cluster sampling design; Fasting samples |
| 39 | Siziya^31^ | Zambia | Population based survey (STEPS) | Kitwe (urban); men & women aged 25-92 | 2010 | 1627 | WC, BMI | BP | Multistage cluster sampling design |
| 40 | Siziya^32^ | Zambia | Population based survey (STEPS) | Lusaka (urban); men & women aged 25-90 | 2008 | 1928 | WC, BMI | BP, glucose, TC | Multistage cluster sampling design; Fasting samples |
| 41 | Chifamba^33^ | Zimbabwe | Population based survey | Marondera (urban); mean & women aged ≥25 years | Not provided | 177 | WC, BMI | BP, glucose, HDL-C, LDL-C, TG, TC | Fasting samples |
|  |  |  |  |  | **TOTAL** | **86 354** |  |  |  |

‡Included in the current analysis

Abbreviations: WC waist circumference (cm); BMI body mass index (kg/m^2^); Hip hip circumference (cm); WHR waist-to-hip ratio; BP blood pressure (systolic/ diastolic) (mmHg); TC total cholesterol (mmol/L); TG triglycerides (mmol/L), HDL-C high-density lipoprotein cholesterol (mmol/L); LDL-C low-density lipoprotein cholesterol (mmol/L); FG/FBG/FPG fasting blood/plasma glucose (mmol/L); HbA1c glycated haemoglobin (%); OGTT oral glucose tolerance test; VLDL very low-density lipoprotein.

**References**

1. Delisle H, Ntandou-Bouzitou G, Agueh V, Sodjinou R, Fayomi B. Urbanisation, nutrition transition and cardiometabolic risk: the Benin study. *Br J Nutr* 2012; **107**(10): 1534-44.

2. Kamadjeu RM, Edwards R, Atanga JS, Unwin N, Kiawi EC, Mbanya JC. Prevalence, awareness and management of hypertension in Cameroon: findings of the 2003 Cameroon Burden of Diabetes Baseline Survey. *J Hum Hypertens* 2006; **20**(1): 91-2.

3. Nasila Sungwacha J, Tyler J, Longo-Mbenza B, Lasi On'Kin JB, Gombet T, Erasmus RT. Assessing clustering of metabolic syndrome components available at primary care for Bantu Africans using factor analysis in the general population. *BMC Res Notes* 2013; **6**: 228.

4. Longo-Mbenza B, Efini B, Ekwanzala, Ngoma V, Nahimana D, Fuele M, et al. Survey on risk factors for Non Communicable Diseases in Kinshasa , Capital of DR Congo according to the WHO STEPs approach. 2006.

5. Muyer MT, Muls E, Mapatano MA, Makulo JR, Mvitu M, Kimenyembo W, et al. Diabetes and intermediate hyperglycaemia in Kisantu, DR Congo: a cross-sectional prevalence study. *BMJ Open* 2012; **2**(6).

6. Christensen DL, Eis J, Hansen AW, Larsson MW, Mwaniki DL, Kilonzo B, et al. Obesity and regional fat distribution in Kenyan populations: impact of ethnicity and urbanization. *Ann Hum Biol* 2008; **35**(2): 232-49.

7. Wesseh C, Clement P, Cowan M. Ministry of Health and Social Welfare, Republic of Liberia. Liberia Chronic Disease Risk Factor Surveillance, 2011, Report.

8. Isezuo SA, Sabir AA, Ohwovorilole AE, Fasanmade OA. Prevalence, associated factors and relationship between prehypertension and hypertension: a study of two ethnic African populations in Northern Nigeria. *J Hum Hypertens* 2011; **25**(4): 224-30.

9. Okafor CI, Fasanmade O, Ofoegbu E, Ohwovoriole AE. Comparison of the performance of two measures of central adiposity among apparently healthy Nigerians using the receiver operating characteristic analysis. *Indian J Endocrinol Metab* 2011; **15**(4): 320-6.

10. Gezawa ID, Puepet FH, Mubi BM, Uloko AE, Bakki B, Talle MA, et al. Prevalence of overweight and obesity in Maiduguri, North-Eastern Nigeria. *Niger J Med* 2013; **22**(3): 171-4.

11. Egbe EO, Asuquo OA, Ekwere EO, Olufemi F, Ohwovoriole AE. Assessment of anthropometric indices among residents of Calabar, South-East Nigeria. *Indian J Endocrinol Metab* 2014; **18**(3): 386-93.

12. Bovet P, Shamlaye C, Kitua A, Riesen WF, Paccaud F, Darioli R. High prevalence of cardiovascular risk factors in the Seychelles (Indian Ocean). *Arterioscler Thromb* 1991; **11**(6): 1730-6.

13. Bovet P, Perret F, Shamlaye C, Darioli R, Paccaud F. The Seychelles Heart Study II: methods and basic findings. *Seychelles Medical and Dental Journal* 1997; **1997**(1): 8-24.

14. Bovet P, Shamlaye C, Gabriel A, Riesen W, Paccaud F. Prevalence of cardiovascular risk factors in a middle-income country and estimated cost of a treatment strategy. *BMC Public Health* 2006; **6**: 9.

15. Bovet P, Viswanathan B, Louange M, Gedeon J. Ministry of Health, Republic of Seychelles. National Survey of Noncommunicable Diseases in Seychelles, 2013-2014 (Seychelles Heart Study IV) : methods and main findings. 2015.

16. Motala AA, Esterhuizen T, Gouws E, Pirie FJ, Omar MA. Diabetes and other disorders of glycemia in a rural South African community: prevalence and associated risk factors. *Diabetes Care* 2008; **31**(9): 1783-8.

17. Crowther NJ, Norris SA. The current waist circumference cut point used for the diagnosis of metabolic syndrome in sub-Saharan African women is not appropriate. *PLoS One* 2012; **7**(11): e48883.

18. Mollentze WF, Moore AJ, Steyn AF, Joubert G, Steyn K, Oosthuizen GM, et al. Coronary heart disease risk factors in a rural and urban Orange Free State black population. *S Afr Med J* 1995; **85**(2): 90-6.

19. Thorogood M, Connor M, Tollman S, Lewando Hundt G, Fowkes G, Marsh J. A cross-sectional study of vascular risk factors in a rural South African population: data from the Southern African Stroke Prevention Initiative (SASPI). *BMC Public Health* 2007; **7**: 326.

20. Sanet van Zyl, Lynette J. van der Merwe, Walsh CM, Groenewald AJ, Francois C. van Rooyen. Risk-factor profiles for chronic diseases of lifestyle and metabolic syndrome in an urban and rural setting in South Africa. *Afr J Prim Health Care Fam Med* 2012; **4**(1): 346.

21. Tanser F, Hosegood V, Barnighausen T, Herbst K, Nyirenda M, Muhwava W, et al. Cohort Profile: Africa Centre Demographic Information System (ACDIS) and population-based HIV survey. *Int J Epidemiol* 2008; **37**(5): 956-62.

22. Hird T, Young E, Pirie F, Riha J, Esterhuizen T, O’Leary B, et al. Study profile: the Durban Diabetes Study (DDS): a platform for chronic disease research. *Global Health, Epidemiology and Genomics* 2016; **1**(2).

23. Teo K, Chow CK, Vaz M, Rangarajan S, Yusuf S. The Prospective Urban Rural Epidemiology (PURE) study: examining the impact of societal influences on chronic noncommunicable diseases in low-, middle-, and high-income countries. *Am Heart J* 2009; **158**(1): 1-7.e1.

24. van Rooyen JM, Kruger HS, Huisman HW, Wissing MP, Margetts BM, Venter CS, et al. An epidemiological study of hypertension and its determinants in a population in transition: the THUSA study. *J Hum Hypertens* 2000; **14**(12): 779-87.

25. Njelekela MA, Mpembeni R, Muhihi A, Mligiliche NL, Spiegelman D, Hertzmark E, et al. Gender-related differences in the prevalence of cardiovascular disease risk factors and their correlates in urban Tanzania. *BMC Cardiovasc Disord* 2009; **9**: 30.

26. Walker R, Whiting D, Unwin N, Mugusi F, Swai M, Aris E, et al. Stroke incidence in rural and urban Tanzania: a prospective, community-based study. *Lancet Neurol* 2010; **9**(8): 786-92.

27. Dewhurst MJ, Dewhurst F, Gray WK, Chaote P, Orega GP, Walker RW. The high prevalence of hypertension in rural-dwelling Tanzanian older adults and the disparity between detection, treatment and control: a rule of sixths? *J Hum Hypertens* 2013; **27**(6): 374-80.

28. Kokou A, Séraphin AK, Kossivi A, Kossi A, Dégnon A, Kwami A, et al. Final Report of the STEPS survey 2010, Ministry of Health , Togo. 2012.

29. Asiki G, Murphy G, Nakiyingi-Miiro J, Seeley J, Nsubuga RN, Karabarinde A, et al. The general population cohort in rural south-western Uganda: a platform for communicable and non-communicable disease studies. *Int J Epidemiol* 2013; **42**(1): 129-41.

30. Besa C, Mulenga D, Babaniyi O, Songolo P, Muula AS, Rudatsikira E, et al. Overweight and Obesity in Kaoma and Kasama Rural Districts of Zambia: Prevalence and Correlates in 2008-2009 Population Based Surveys. *J Hypertens* 2013; **2**(1).

31. Siziya S, Rudatsikira E, Babaniyi O, Songolo P, Mulenga D, Muula AS. Prevalence and Correlates of Hypertension among Adults Aged 25 Years or Older in a Mining Town of Kitwe, Zambia. *J Hypertens* 2012; **1**(3).

32. Rudatsikira E, Muula AS, Mulenga D, Siziya S. Prevalence and correlates of obesity among Lusaka residents, Zambia: a population-based survey. *Int Arch Med* 2012; **5**: 14.

33. Mufunda J, Scott LJ, Chifamba J, Matenga J, Sparks B, Cooper R, et al. Correlates of blood pressure in an urban Zimbabwean population and comparison to other populations of African origin. *J Hum Hypertens* 2000; **14**(1): 65-73.
