# Supplementary material for: Deriving an optimal threshold of waist circumference for detecting cardiometabolic risk in sub-Saharan Africa
Source: Int J Obes (Lond). 2017 Oct 31;42(3):487–94. doi: 10.1038/ijo.2017.240 (PMC5880575; doi:10.1038/ijo.2017.240)
Supplement: Supplementary Table 2 [file ijo2017240x2.docx]

**Table S2. Details of anthropometry and blood pressure measurements**

| **Study** | **Anthropometry** | | | | | **BP measurement** | | | **Blood samples** |
| --- | --- | --- | --- | --- | --- | --- | --- | --- | --- |
|  | Weight: measured with participants bare foot and in light clothing using a calibrated digital scale to the nearest 100g. | Height: measured with a stadiometer to the nearest 0.1 cm. | Waist and Hip circumference measured with a flexible, non-elastic tape, held parallel to the ground. | Waist circumference: The smallest circumference to the nearest 0.1 cm, between the xiphisternum and the umbilicus on expiration while standing, was taken as the waist circumference. | Hip Circumference: taken at the maximum posterior protuberance of the buttocks to the nearest 0.1 cm. | What machine was used? 1=Omron BP monitor 2= standard mercury sphygmomanometer 3=Other | Three BP measurements at 2-min intervals after the participant had been seated for 5 min. The average of the second and third BP measurements was used in the analysis. | Blood pressure taken with an appropriately sized cuff. | Blood for glucose and lipid measurement were drawn following an overnight 8-10 h fast. |
| Delisle (Benin) | Mechanical scale | Yes | Only waist circumference measured |  |  | 2 | Average of 2 measures | Yes | Yes |
| Nzambi (DR Congo) |  |  | - | - | - | - | - | - | - |
| Longo-Mbenza (DR Congo) | - | - | - | - | - | - | - | - | - |
| Christensen (Kenya) | Yes |  | Yes | Measured midway between the iliac crest and the costal margin | Measured by using the trochanter major as the fix-point! | 1 | Two measurements were done, a third was only done if SBP deviated by more than 5 mmHg between the first two. Average of 2 or 3 taken | Yes | Yes (venous blood used) |
| Oladapo-Lola (Nigeria) | Yes | Yes | Yes | Yes | Yes | 1 | Yes | Yes | Yes |
| Bovet (Seychelles) | Yes | Yes | Yes | 1989: 2 in 1989, 1994; 1 in 2004, 2013 |  | 1 | Yes |  |  |
| Motala (South Africa) | Yes | Yes | Yes | Mechanical scale | Yes | 2 | Yes | Yes | Yes |
| Crowther (South Africa) | - | - | - | - | - | - | - | - | - |
| Mollentze (South Africa) | Used a calibrated Seca balance scale | Yes | Yes | To nearest 0.5 cm measured halfway between the 12th rib and superior iliac crest with the patient standing, at full expiration and arms at the sides. Only two observers did measurements. | To nearest 0.5 cm. Only two observers did all measurements. | 3. Hawskley random zero. | Two BP measurements, lying down, after 5 min rest. The set of values with the lowest diastolic measurement was used (Only two observers). | Yes | Yes |
| Walsh (South Africa -Rural) | Yes | Yes | Yes | Yes | Yes | 1 | Taken in supine position | Yes | Yes |
| Walsh (South Africa -Urban) | Yes | Yes | Yes | Yes | Yes | 1 | Taken in supine position | Yes | Yes |
| Durban Diabetes Study (South Africa) | Yes | Yes | Yes | Yes | Yes | 1 | Yes | Yes | Yes |
| Schutte (South Africa) | Yes | Yes | Yes | Yes | Yes | 1 | After a 10-minute rest period, BP was measured in duplicate (5 minutes apart) on the right upper arm, while the participants were seated upright with the right arm supported at heart level. Second BP was used. | Yes | Yes |
| Kruger (South Africa) | Yes | Yes | Yes | Yes | Yes | 2. standard mercury sphygmomanometer, model ALPK2, Tycos, Arden, NC, USA | Two BP measurements at 2-min intervals after the participant had been seated for 5 min. The second BP measurement was used in the analysis | A standard mercury sphygmomanometer with an appropriately sized cuff was used to take two BP measurements at 2-min intervals after the participant had been seated for 5 min. The second BP measurement was used in the analysis. | Yes |
| Njelekela (Tanzania) | Yes . Body weight was measured with subject standing and wearing light clothes and without shoes to the nearest 0.1 kg using a digital scale (Tanita, Tokyo, Japan). | Height was measured to the nearest 0.5 cm using a portable stadiometer. | Yes | Hip circumference (HC) and waist circumference (WC) were measured using standardized procedures to the nearest 0.5 cm using a flexible tape measure. | Hip circumference (HC) and waist circumference (WC) were measured using standardized procedures to the nearest 0.5 cm using a flexible tape measure. | 1 (Omron Digital HEM-907, Tokyo, Japan). | Three blood pressure readings were taken on the left upper arm with the participant in a seated position after at least 5 to 10 minutes of rest. The average of the three readings was included in analyses. | Yes | Blood samples were taken after at least 10 hours of fasting. |
| Unwin (Tanzania) | Weight measured to the nearest 0.1 kg using Mechanical scale | Height was measured on a stadiometer to the nearest 0.5 cm | Yes | No, Bony landmarks were used: mid axillary line, mid-point between iliac crest and lower costal margin, and to the nearest centimetre. It was measured twice with the mean of the two measurements being used. The other details are the same i.e. standing and in gentle expiration. | No, Measured twice to the nearest centimetre, and the mean of the two measurements used. | Omron M4 used | Seated for at least 5 mins, and then 5 mins between measurements. Only two measurements were taken, and the mean of the two used. I can't remember why this was done, but agreed that 3 would have been better and discard the 1st | Yes - appropriately sized cuff | Yes |
| GPC, General Population Cohort, (Uganda) | Yes | Yes | Yes | Yes | Yes | 1 | Yes | Yes | Non-fasting samples only |

- Information not provided
