# Supplementary material for: Deriving an optimal threshold of waist circumference for detecting cardiometabolic risk in sub-Saharan Africa
Source: Int J Obes (Lond). 2017 Oct 31;42(3):487–94. doi: 10.1038/ijo.2017.240 (PMC5880575; doi:10.1038/ijo.2017.240)
Supplement: Supplementary Table 3 [file ijo2017240x3.docx]

**Table S3. Positive predictive value of derived waist circumference and prevalence of at least two components of metabolic syndrome in individual studies included in the validation dataset (N 4301: Men 1674, Women 2627).**

| Study | Prevalence of at least two components of MS, men | Positive predictive value (PPV) | Prevalence of at least two components of MS, women | Positive predictive value (PPV) |
| --- | --- | --- | --- | --- |
| Delisle (Benin) | 10.0% | 19% | 15.2% | 21% |
| Christensen (Kenya) | 19.5% | 38% | 21.8% | 37.4 |
| Nzambi (DR Congo) | 22.9% | 28.40% | 35.6% | 36.90% |
| Motala (South Africa) | 24.9% | 37.00% | 38.2% | 47.00% |
| Walsh (South Africa - Rural) | 48.3% | 75.80% | 72.2% | 80.80% |
