# Supplementary material for: Deriving an optimal threshold of waist circumference for detecting cardiometabolic risk in sub-Saharan Africa
Source: Int J Obes (Lond). 2017 Oct 31;42(3):487–94. doi: 10.1038/ijo.2017.240 (PMC5880575; doi:10.1038/ijo.2017.240)
Supplement: Supplementary Table 4 [file ijo2017240x4.docx]

**Table S4. Participant characteristics by sex in the derivation dataset (N 19 880: 8055, Women 11 825).**

| **Characteristic*** | N† (Men/Women) | Men | Women | All |
| --- | --- | --- | --- | --- |
| **Mean** |  |  |  |  |
| Age (years) | 8055/11 825 | 41.2 (40.8-41.5) | 41.8 (41.6-42.1) | 41.6 (41.3-41.8) |
| WC | 8055/11 825 | 79.0 (78.8-79.2) | 82.2 (82.0-82.5) | 80.9 (80.7-81.1) |
| BMI | 8039/11 802 | 22.2 (22.1-22.3) | 25.4 (25.3-25.6) | 24.1 (24.1-24.2) |
| Hip | 7861/11 644 | 88.7 (88.5-89.0) | 97.7 (97.4-98.0) | 94.1 (3.9-94.4) |
| WHR | 7861/11 644 | 0.89 (0.89-0.90) | 0.85 (0.85-0.85) | 0.87 (0.87-.87) |
| WHtR | 7248/10 604 | 0.48 (0.48-0.48) | 0.53 (0.53-0.53) | 0.51 (0.51-0.51) |
| SBP | 8052/11 821 | 127 (127-128) | 125 (125-125) | 126 (125-126) |
| DBP | 8054/11 823 | 78 (77-78) | 78 (78-78) | 78 (78-78) |
| TC | 6222/8795 | 4.0 (4.0-4.1) | 4.3 (4.3-4.3) | 4.2 (4.2-4.2) |
| TG**^‡^** | 8055/11 825 | 1.04 (1.03-1.06) | 1.03 (1.02-1.04) | 1.03 (1.03-1.04) |
| HDL | 8055/11 825 | 1.2 (1.2-1.2) | 1.2 (1.2-1.2) | 1.2 (1.2-1.2) |
| LDL | 6080/9120 | 2.2 (2.2-2.2) | 2.4 (2.4-2.4) | 2.3 (2.3-2.3) |
| FG | 5091/ 8016 | 5.3 (5.2-5.3) | 5.4 (5.3-5.4) | 5.3 (5.3-5.4) |
| HbA1c | 3934/5691 | 5.3 (5.2-5.3) | 5.4 (5.4-5.4) | 5.4 (5.3-5.4) |
| **Prevalence** |  |  |  |  |
| MS(≥2 of 5 abnormalities) ^##^ | 8055/11 825 | 31 (30-32) | 39 (38-39) | 35 (35-36) |
| WC ≥94/80 (men/women) ^##^ | 8055/11 825 | 12 (12-13) | 50 (49-51) | 35 (34-36) |
| BMI ≥25 ^##^ | 8039/11 802 | 21 (20-22) | 43 (42-44) | 34 (33-35) |
| BMI≥30 ^##^ | 8039/11 802 | 6 (6-7) | 21 (21-22) | 15 (15-16) |
| WHR >1.0/0.85 (men/women) ^##^ | 7861/11 644 | 11 (11-12) | 46 (45-47) | 32 (31-32) |
| WHtR >0.5 ^##^ | 7248/10 604 | 28 (7-29) | 56 (55-57) | 45 (44-45) |
| BP ≥130/85 or use of anti-hypertensive medication ^##^ | 8055/11 825 | 44 (43-45) | 39 (39-40) | 41 (41-42) |
| TC >5.0 ^##^ | 6222/ 8795 | 22 (21-23) | 26 (25-27) | 24 (24-25) |
| TG >1.7 | 8055/11 825 | 14 (14-15) | 14 (13-15) | 14 (14-15) |
| HDL-C <1.0/1.3 (men/women )^##^ | 8055/11 825 | 38 (37-39) | 66 (65-67) | 55 (54-56) |
| LDL-C >3.0 ^##^ | 6080 / 9120 | 18 (17-19) | 23 (23-24) | 21 (21-22) |
| FG >5.6 or HbA1c≥5.7 **^**^** | 8055/11 825 | 20 (19-20) | 18 (18-19) | 19 (18-19) |
| Ever smoked ^##^ | 6052/7894 | 26 (24-27) | 5 (5-6) | 14 (13-14) |
| Ever consumed alcohol ^##^ | 3679/ 4877 | 57 (55-58) | 46 (44-47) | 50 (49-51) |
| Data are mean (95% CI) (except as indicated by **^‡^**) and prevalence (%) (95% CI) (Some CI limits coincide due to rounding errors). Notes: *****Means and prevalence are standardised to the WHO world standard population using the direct method; †The total of men and women for some characteristics is less than 19880 because of missing data; **^‡^**Data are median standardised to the median age in the full dataset; **^**^**Individuals with both FG and HbA1c measurements available were classified using FG. Abbreviations: N number of participants; CI confidence interval, WC waist circumference (cm); BMI body mass index (kg/m^2^); Hip hip circumference (cm); WHR waist-to-hip ratio, WHtR waist-to-height ratio; SBP systolic blood pressure (mmHg); DBP diastolic blood pressure (mmHg), BP blood pressure (mmHg); TC total cholesterol (mmol/L); TG triglycerides (mmol/L), HDL-C high-density lipoprotein cholesterol (mmol/L); LDL-C low-density lipoprotein cholesterol (mmol/L); FG fasting blood/plasma glucose (mmol/L); HbA1c glycated haemoglobin (%). ^##^ P <0.001 (comparisons are between men and women). | | | | |
