# Supplementary material for: Deriving an optimal threshold of waist circumference for detecting cardiometabolic risk in sub-Saharan Africa
Source: Int J Obes (Lond). 2017 Oct 31;42(3):487–94. doi: 10.1038/ijo.2017.240 (PMC5880575; doi:10.1038/ijo.2017.240)
Supplement: Supplementary Table 5 [file ijo2017240x5.docx]

**Table S5. Participant characteristics by sex in the validation dataset (N 4301: Men 1674, Women 2627).**

| **Characteristic*** | N† (Men/Women) | Men | Women | All |
| --- | --- | --- | --- | --- |
| **Mean** |  |  |  |  |
| Age (years) | 1674/ 2627 | 42.9 (42.2-43.6) | 43.9 (43.3-44.4) | 43.5 (43.1-43.9) |
| WC | 1674/2627 | 79.3 (78.3-79.9) | 82.0 (81.5-82.5) | 80.1 (80.6-81.4) |
| BMI | 1670/2624 | 21.4 (21.1-21.6) | 24.5 (24.2-24.7) | 23.3 (23.1-23.4) |
| Hip | 1373/2289 | 89.9 (89.3-90.5) | 97.1 (96.5-97.6) | 94.4 (94.0-94.9) |
| WHR | 1373/2289 | 0.88 (0.87-0.89) | 0.84 (0.84-0.84) | 0.85 (0.85-.86) |
| WHtR | 1671/2626 | 0.47 (0.46-0.47) | 0.51 (0.51-0.52) | 0.50 (0.49-0.50) |
| SBP | 1483/2452 | 111 (109-113) | 114 (112-116) | 113 (112-114) |
| DBP | 1649/2609 | 76 (75-6) | 78 (77-78) | 77 (77-77) |
| TC | 1674/2627 | 4.0 (3.9-4.1) | 4.0 (4.0-4.1) | 4.0 (4.0-4.0) |
| TG**^‡^** | 1674/2627 | 0.86 (0.84-0.88) | 0.85 (0.83-0.87) | 0.86 (0.84-0.87) |
| HDL | 1674/2627 | 1.1 (1.0-1.1) | 1.1 (1.1-1.1) | 1.1 (1.1-1.1) |
| LDL | 608/1365 | 2.5 (2.4-2.5) | 2.5 (25-26) | 2.5 (2.5-2.5) |
| FG | 1602/2555 | 4.6 (4.6-4.7) | 4.8 (4.7-4.8) | 4.7 (4.7-4.8) |
| HbA1c | 139/160 | 5.0 (4.9-5.2) | 5.4 (5.1-5.7) | 5.2 (5.0-5.4) |
| **Prevalence** |  |  |  |  |
| MS(≥2 of 5 components) ^##^ | 1673 /2627 | 20 (18-22) | 32 (30-33) | 27 (26-29) |
| WC ≥94/80 (men/women) ^##^ | 1674 /2627 | 10 (9-12) | 47 (45-49) | 33 (32-35) |
| BMI ≥25 ^##^ | 1670/2624 | 15 (13-16) | 36 (34-38) | 28 (26-29) |
| BMI ≥30 ^##^ | 1670/2624 | 5 (3-6) | 16 (15-18) | 12 (11-13) |
| WHR >1.0/0.85 (men/women) ^##^ | 1373/2289 | 4 (3-5) | 39 (37-41) | 26 (24-27) |
| WHtR >0.5 ^##^ | 1671/2626 | 25 (23-27) | 47 (45-49) | 38 (37-40) |
| BP ≥130/85 or use of anti-hypertensive medication ^#^ | 1674/2627 | 33 (31-36) | 38 (36-39) | 36 (35-38) |
| TC >5.0 | 1674/2627 | 16 (14-18) | 17 (16-19) | 17 (16-18) |
| TG >1.7 ^#^ | 1674/2627 | 6 (5-8) | 9 (8-10) | 8 (7-9) |
| HDL-C <1.0/1.3 (men/women) ^##^ | 1674/2627 | 48 (46-1) | 71 (69-73) | 62 (61-64) |
| LDL-C >3.0 ^#^ | 608/1365 | 26 (23-29) | 27 (25-29) | 27 (25-29) |
| FG >5.6 or HbA1c ≥5.7 ****** | 1674/2627 | 7 (5-8) | 8 (7-9) | 7 (6-8) |
| Ever smoked ^##^ | 746/1506 | 17 (14-20) | 7 (6-8) | 10 (9-12) |
| Ever consumed alcohol ^##^ | 744/1490 | 26 (23-29) | 5 (4-7) | 12 (11-13) |
| Data are mean (95% CI) (except as indicated by **^‡^**) and prevalence (%) (95% CI) (Some CI limits coincide due to rounding errors). Notes: *****Means and prevalence are standardised to the WHO world standard population using the direct method; †The total of men and women for some characteristics is less than 4301 because of missing data; **^‡^**Data are median standardised to the median age in the full dataset; ******Individuals with both FG and HbA1c measurements available were classified using FG. Abbreviations: N number of participants; CI confidence interval, WC waist circumference (cm); BMI body mass index (kg/m^2^); Hip hip circumference (cm); WHR waist-to-hip ratio, WHtR waist-to-height ratio; SBP systolic blood pressure (mmHg); DBP diastolic blood pressure (mmHg), BP blood pressure (mmHg); TC total cholesterol (mmol/L); TG triglycerides (mmol/L), HDL-C high-density lipoprotein cholesterol (mmol/L); LDL-C low-density lipoprotein cholesterol (mmol/L); FG fasting blood/plasma glucose (mmol/L); HbA1c glycated haemoglobin (%). ^##^ P <0.001, ^#^ P <0.05, (comparisons are between men and women). | | | | |
