# Supplementary material for: Deriving an optimal threshold of waist circumference for detecting cardiometabolic risk in sub-Saharan Africa
Source: Int J Obes (Lond). 2017 Oct 31;42(3):487–94. doi: 10.1038/ijo.2017.240 (PMC5880575; doi:10.1038/ijo.2017.240)
Supplement: Supplementary Table 6 [file ijo2017240x6.docx]

**Table S6. Characteristics of receiver operating characteristic curve analyses for the derivation of anthropometric cut-points for identifying raised blood pressure and components of dyslipidaemia in the derivation dataset (N 19 880: Men 8055, Women 11 825).**

|  | Men | | | | | | Women | | | | | |
| --- | --- | --- | --- | --- | --- | --- | --- | --- | --- | --- | --- | --- |
| Index | AUC(95%CI) | P-value* for difference in AUC between index and WC | Cut-point (95%CI) | Sensitivity (95%CI) | Specificity (95%CI) | Youden index | AUC(95%CI) | p-value* for difference in AUC between index and WC | Cut-point (95%CI) | Sensitivity (95%CI) | Specificity (95%CI) | Youden index |
| **Raised BP** |  |  |  |  |  |  |  |  |  |  |  |  |
| WC | 0.67(066-0.68) |  | 81.1 (79.1-83.1) | 49 (48-51) | 77 (76-78) | 0.261 | 0.66 (0.65-0.67) |  | 84.0 (81.6-86.5) | 57 (55-58) | 71 (70-72) | 0.272 |
| BMI | 0.62(0.60-0.63) | <0.001 | 23.4 (22.2-24.6) | 42 (40-43) | 78 (77-79) | 0.194 | 0.64 (0.63-0.65) | <0.001 | 25.7 (24.9-26.5) | 56 (54-57) | 68 (67-69) | 0.236 |
| WHR | 0.58(0.57-0.60) | <0.001 | 0.88 (0.87-0.89) | 60 (59-62) | 55 (54-57) | 0.155 | 0.54 (0.53-0.55) | <0.001 | 0.84 (0.80-0.87) | 57 (55-58) | 52 (50-53) | 0.081 |
| WHtR | 0.63(0.62-0.64) | <0.001 | 0.48 (0.46-0.50) | 50 (48-52) | 72 (71-74) | 0.220 | 0.64 (0.63-0.65) | 0.003 | 0.53 (0.51-0.54) | 60 (58-61) | 64 (63-65) | 0.237 |
|  |  |  |  |  |  |  |  |  |  |  |  |  |
| **Raised TG** |  |  |  |  |  |  |  |  |  |  |  |  |
| WC | 0.68(0.66-0.69) |  | 80.6 (78.1-83.0) | 61 (58-64) | 67 (66-68) | 0.284 | 0.63 (0.62-0.65) |  | 81.2 (77.3-85.1) | 66 (64-69) | 54 (53-55) | 0.206 |
| BMI | 0.65(0.63-0.66) | <0.001 | 22.7 (21.2-24.3) | 56 (53-59) | 68 (66-69) | 0.232 | 0.59 (0.58-0.60) | <0.001 | 24.8 (22.7-26.9) | 59 (57-61) | 55 (54-56) | 0.138 |
| WHR | 0.64(0.63-0.66) | <0.001 | 0.89 (0.87-0.91) | 63 (60-66) | 59 (58-60) | 0.220 | 0.62 (0.60-0.63) | 0.018 | 0.84 (0.83-0.85) | 67 (65-69) | 52 (51-53) | 0.189 |
| WHtR | 0.68(0.67-0.70) | 0.596 | 0.48 (0.47-0.49) | 67 (64-69) | 63 (61-64) | 0.291 | 0.64 (0.63-0.65) | <0001 | 0.54 (0.53-0.55) | 60 (57-62) | 62 (61-63) | 0.216 |
|  |  |  |  |  |  |  |  |  |  |  |  |  |
| **Low HDL-C** |  |  |  |  |  |  |  |  |  |  |  |  |
| WC | 0.44(0.43-0.46) |  | 108.7 (98.2-119.2) | 3 (2-4) | 98 (97-98) | 0.007 | 0.50 (0.49-0.51) |  | 71.0(54.8-87.2) | 79 (78-80) | 23 (22-24) | 0.02 |
| BMI | 0.48(0.46-0.49) | <0.001 | 33.2 (21.9-44.5) | 3 (2-4) | 98 (97-98) | 0.007 | 0.50 (0.49-0.51) | 0.449 | 29.8(21.4-38.3) | 25 (24-26) | 78 (76-79) | 0.023 |
| WHR | 0.45(0.44-0.46) | 0.352 | 0.77 (0.59-0.95) | 97 (96-97) | 4 (3-5) | 0.008 | 0.48 (0.47-0.49) | <0.001 | 0.77(0.46-1.08) | 79 (79-80) | 21 (20-23) | 0.007 |
| WHtR | 0.48(0.47-0.50) | <0.001 | 0.41 (0.40-0.42) | 93 (92-94) | 10 (9-11) | 0.033 | 0.51 (0.50-0.53) | 0.078 | 0.45(0.40-0.50) | 85 (84-86) | 19 (18-20) | 0.037 |
|  |  |  |  |  |  |  |  |  |  |  |  |  |
| **Raised TC** |  |  |  |  |  |  |  |  |  |  |  |  |
| WC | 0.73(0.71-0.74) |  | 80.9 (79.1-82.7) | 67 (65-70) | 71 (70-72) | 0.381 | 0.65 (0.64-0.66) |  | 83.5 (82.2-84.9) | 61 (59-63) | 65 (64-66) | 0.261 |
| BMI | 0.69(0.68-0.71) | <0.001 | 23.4 (22.7-24.2) | 57 (54-59) | 76 (75-77) | 0.324 | 0.66 (0.65-0.67) | 0.057 | 25.6 (24.5-26.9) | 61 (59-63) | 66 (65-67) | 0.268 |
| WHR | 0.69(0.67-0.70) | <0.001 | 0.87 (0.85-0.89) | 74 (72-76) | 55 (53-56) | 0.286 | 0.59 (0.57-0.60) | <0.001 | 0.86 (0.84-0.88) | 47 (46-49) | 68 (66-69) | 0.149 |
| WHtR | 0 .70(0.68-0.71) | <0.001 | 0.51 (0.49-0.52) | 54 (52-57) | 79 (78-80) | 0.333 | 0.65 (0.64-0.66) | 0.022 | 0.53 (0.52-0.54) | 63 (61-65) | 62 (61-64) | 0.249 |
|  |  |  |  |  |  |  |  |  |  |  |  |  |
| **Raised LDL-C** |  |  |  |  |  |  |  |  |  |  |  |  |
| WC | 0.71(0.69-0.73) |  | 79.5 (77.2-81.9) | 60 (57-62) | 76 (75-77) | 0.355 | 0.63 (0.62-0.65) |  | 82.6 (80.5-84.7) | 57 (55-59) | 66 (65-67) | 0.228 |
| BMI | 0.67(0.66-0.69) | <0.001 | 22.6 (21.8-23.5) | 52 (49-55) | 77 (76-78) | 0.295 | 0.63 (0.62-0.65) | 0.956 | 24.9 (23.6-26.2) | 57 (55-59) | 66 (64- 67) | 0.224 |
| WHR | 0.66(0.64-0.68) | <0.001 | 0 .88 (0.87-0.89) | 64 (61-66) | 63 (62-65) | 0.268 | 0.55 (0.53-0.56) | <0.001 | 0.84 (0.79-0.89) | 54 (52-56) | 54 (53-56) | 0.085 |
| WHtR | 0.70(0.68-0.72) | <0.001 | 0.49 (0.47-0.50) | 55 (52-59) | 79 (77-80) | 0.339 | 0.63 (0.61-0.64) | 0.322 | 0.52 (0.51-0.54) | 61 (59-64) | 61 (60-62) | 0.223 |
|  |  |  |  |  |  |  |  |  |  |  |  |  |
| **Raised FG/HbA1c** |  |  |  |  |  |  |  |  |  |  |  |  |
| WC | 0.61(0.60-0.63) |  | 86.7 (83.4-89.6) | 41 (39-44) | 81 (80-82) | 0.223 | 0.60 (0.59-0.61) |  | 87.9 (83.8-91.9) | 48 (46-50) | 70 (69-71) | 0.178 |
| BMI | 0.61(0.60-0.63) | 0.561 | 23.8 (22.3-25.2) | 44 (41-46) | 75 (74-76) | 0.189 | 0.58 (0.57-0.59) | <0.001 | 27.0 (24.9-29.0) | 47 (45-49) | 67 (66-68) | 0.139 |
| WHR | 0.61(0.60-0.63) | 0.253 | 0.89 (0.87-0.90) | 60 (58-63) | 58 (57-60) | 0.188 | 0.61 (0.60-0.62) | 0.562 | 0.88 (0.86-0.90) | 50 (48-52) | 68 (67-69) | 0.18 |
| WHtR | 0.64(0.62-0.65) | <0.001 | 0.51 (0.49-0.53) | 46 (43-48) | 78 (77-79) | 0.232 | 0.64 (0.63-0.65) | 0.710 | 0.56 (0.53-0.58) | 54 (52-56) | 68 (67-69) | 0.223 |
| Abbreviations: N Number of participants; AUC area under the curve; BP blood pressure (mmHg); WC waist circumference (cm); BMI body mass index (kg/m2); WHR waist-to-hip ratio; WHtR waist-to-height ratio; TC total cholesterol (mmol/L); TG triglycerides (mmol/L); HDL-C high-density lipoprotein cholesterol (mmol/L); LDL-C low-density lipoprotein cholesterol (mmol/L); FG fasting plasma/blood glucose (mmol/L); HbA1c glycated haemoglobin (%); CI confidence interval; *P-values reported for tests restricted to individuals in which both WC and the anthropometric measure assessed were determined. | | | | | | | | | | | | |
