# Supplementary material for: Deriving an optimal threshold of waist circumference for detecting cardiometabolic risk in sub-Saharan Africa
Source: Int J Obes (Lond). 2017 Oct 31;42(3):487–94. doi: 10.1038/ijo.2017.240 (PMC5880575; doi:10.1038/ijo.2017.240)
Supplement: Supplementary Table 7 [file ijo2017240x7.docx]

**Table S7. Performance of derived waist circumference cut-points for detecting raised blood pressure and components of dyslipidaemia in the validation dataset (N 4301: Men 1674, Women 2627).**

| Anthropometric variable | Derived/Current index | N^†^ | Cut-point | Sensitivity % (95% CI) | Specificity % (95% CI) | Positive predictive value % (95% CI) | Negative predictive value % (95% CI) |
| --- | --- | --- | --- | --- | --- | --- | --- |
| **Men** |  |  |  |  |  |  |  |
| Raised BP | Derived | 1674 | 81.1 | 51 (47-55) | 71 (68-73) | 50 (46-54) | 72 (69-74) |
|  | Current | 1674 | 94.0 | 24 (20-27) | 94 (93-96) | 71 (64-77) | 68 (66-71) |
| Raised TG | Derived | 1674 | 80.6 | 77 (69-83) | 64 (62-66) | 16 (13-19) | 97 (96-98) |
|  | Current | 1674 | 94.0 | 48 (40-57) | 91 (90-92) | 32 (26-39) | 95 (94-96) |
| Lowered HDL-C | Derived | 1674 | 108.7 | 3 (2-4) | 98 (97-99) | 53 (36-69) | 52 (50-55) |
|  | Current | 1674 | 94.0 | 14 (12-17) | 89 (87-91) | 54 (47-61) | 53 (51-56) |
| Raised TC | Derived | 1674 | 80.9 | 60 (54-65) | 66 (63-68) | 28 (25-32) | 88 (86-90) |
|  | Current | 1674 | 94.0 | 28 (23-33) | 91 (90-93) | 42 (35-49) | 85 (83-87) |
| Raised LDL-C | Derived | 608 | 79.5 | 65 (57-72) | 58 (53-63) | 40 (35-46) | 79 (74-84) |
|  | Current | 608 | 94.0 | 28 (21-35) | 90 (86-92) | 52 (42-62) | 74 (70-78) |
| Raised FG/HbA1c | Derived | 1674 | 86.7 | 45 (36-54) | 78 (76-80) | 15 (11-19) | 94 (93-96) |
|  | Current | 1674 | 94.0 | 34 (26-43) | 90 (88-91) | 22 (16-28) | 94 (93-95) |
| **Women** |  |  |  |  |  |  |  |
| Raised BP | Derived | 2627 | 84.0 | 57 (54-60) | 68 (66-71) | 56 (53-59) | 69 (67-71) |
|  | Current | 2627 | 80.0 | 69 (66-71) | 56 (53-58) | 52 (50-55) | 72 (69-74) |
| Raised TG | Derived | 2627 | 81.2 | 81 (76-86) | 53 (51-55) | 17 (15-19) | 96 (95-97) |
|  | Current | 2627 | 80.0 | 84 (80-89) | 49 (47-51) | 16 (14-18) | 97 (95-98) |
| Low HDL-C | Derived | 2627 | 71.0 | 84 (82-85) | 18 (16-21) | 72 (70-74) | 31 (27-36) |
|  | Current | 2627 | 80.0 | 55 (53-57) | 47 (43-51) | 72 (70-74) | 30 (27-32) |
| Raised TC | Derived | 2627 | 83.5 | 64 (59-68) | 62 (60-64) | 29 (27-32) | 87 (85-89) |
|  | Current | 2627 | 80.0 | 75 (71-78) | 51 (49-53) | 27 (25-30) | 90 (87-91) |
| Raised LDL-C | Derived | 1365 | 82.6 | 76 (72-80) | 48 (45-51) | 40 (37-44) | 81 (78-85) |
|  | Current | 1365 | 80.0 | 84 (80-87) | 40 (37-44) | 39 (36-42) | 84 (81-88) |
| Raised FG/HbA1c | Derived | 2627 | 87.9 | 61(54-67) | 71 (69-72) | 16 (14-19) | 95 (94-96) |
|  | Current | 2627 | 80.0 | 79 (73-84) | 48 (46-50) | 13 (11-15) | 96 (95-97) |
| Abbreviations: N number of participants; BP blood pressure (mmHg); TC total cholesterol (mmol/L); TG triglycerides (mmol/L); HDL-C high-density lipoprotein cholesterol (mmol/L); LDL-C low-density lipoprotein cholesterol (mmol/L); FG fasting blood/plasma glucose (mmol/L); HbA1c glycated haemoglobin (%); CI confidence interval. ^†^The total of men and women for some indices is less than 4301 because of missing data. | | | | | | | |
