# Supplementary material for: Deriving an optimal threshold of waist circumference for detecting cardiometabolic risk in sub-Saharan Africa
Source: Int J Obes (Lond). 2017 Oct 31;42(3):487–94. doi: 10.1038/ijo.2017.240 (PMC5880575; doi:10.1038/ijo.2017.240)
Supplement: Supplementary Figure 2 [file ijo2017240x9.docx]

Prevalence

Raised WC (WC=94+, Men; 80+, Women)

0

.2

.4

.6

.8

1

15-19

30-34

45-49

60-64

75-79

90-94

Raised BMI (BMI=25+)

0

.2

.4

.6

.8

1

15-19

30-34

45-49

60-64

75-79

90-94

Raised WHR (WHR>1.0, Men; 0.85, Women)

0

.2

.4

.6

.8

1

15-19

30-34

45-49

60-64

75-79

90-94

Raised WHtR (WHtR>0.5)

0

.2

.4

.6

.8

1

15-19

30-34

45-49

60-64

75-79

90-94

Raised BP (BP=130+/85+)

0

.2

.4

.6

.8

1

15-19

30-34

45-49

60-64

75-79

90-94

Raised TG (TG>1.7)

0

.2

.4

.6

.8

1

15-19

30-34

45-49

60-64

75-79

90-94

Lowered HDL (HDL< 1.0, Men; 1.3, Women)

0

.2

.4

.6

.8

1

15-19

30-34

45-49

60-64

75-79

90-94

Raised FG/HbA1c (FG>5.6/HbA1c>5.7)

0

.2

.4

.6

.8

1

15-19

30-34

45-49

60-64

75-79

90-94

Age (Years)

Men

Women

Abbreviations: WC waist circumference; BMI body mass index; WHR waist-hip ratio; BP blood pressure; TG triglycerides; HDL high-density lipoprotein cholesterol; FG fasting plasma/blood glucose; HbA1c glycated haemoglobin.

**Figure S2.** Prevalence of anthropometric and biochemical risk factors in the full data set (Number of participants, 24 181: Men 9729, Women 14 452)
