# Supplementary material for: Deriving an optimal threshold of waist circumference for detecting cardiometabolic risk in sub-Saharan Africa
Source: Int J Obes (Lond). 2017 Oct 31;42(3):487–94. doi: 10.1038/ijo.2017.240 (PMC5880575; doi:10.1038/ijo.2017.240)
Supplement: Supplementary Figure 3 [file ijo2017240x10.docx]

Sensitivity

Abbreviations: AUC area under the curve; WC waist circumference; BMI body mass index; WHR waist-hip ratio; WHtR weight to height ratio.

**Figure S3.** Receiver operating characteristic curve (waist circumference compared to other anthropometric measurements) analyses for predicting at least two of four components of metabolic syndrome excluding waist circumference in the derivation dataset (Number of participants, 19 880**:** Men 8055, Women 11 825), in men (left panel) and women (right panel)
