# Supplementary material for: Deriving an optimal threshold of waist circumference for detecting cardiometabolic risk in sub-Saharan Africa
Source: Int J Obes (Lond). 2017 Oct 31;42(3):487–94. doi: 10.1038/ijo.2017.240 (PMC5880575; doi:10.1038/ijo.2017.240)
Supplement: Supplementary Figure 4 [file ijo2017240x11.docx]

**Key**

1 GPC (Uganda)

2 Christensen (Kenya)

3 Schutte (South Africa)

4 Kruger (South Africa)

5 Delisle (Benin)

6 Walsh (South Africa -Urban)

7 Nzambi (DR Congo)

8 Unwin (Tanzania)

9 Longo-Mbenza (DR Congo)

10 Walsh (South Africa -Rural)

11 Motala (South Africa)

12 Durban Diabetes Study (South Africa)

13 Mollentze (South Africa)

14 Njelekela (Tanzania)

15 Oladapo-Lola (Nigeria)

16 Bovet (Seychelles)

1

2

3

4

5

6

7

8

9

10

11

12

13

14

15

16

Optimal cut-point in the derivation

dataset

70

80

90

100

Waist circumference (95% CI) (cm)

0

5

10

15

20

Prevalence of obesity (BMI=30+), %

Study-specific waist circumference cut-point

**Figure S4.** Study-specific waist circumference cut-points for predicting at least two of the other components of metabolic syndrome in men by prevalence of obesity
