# Supplementary material for: Deriving an optimal threshold of waist circumference for detecting cardiometabolic risk in sub-Saharan Africa
Source: Int J Obes (Lond). 2017 Oct 31;42(3):487–94. doi: 10.1038/ijo.2017.240 (PMC5880575; doi:10.1038/ijo.2017.240)
Supplement: Supplementary Figure 5 [file ijo2017240x12.docx]

**Key**

1 GPC (Uganda)

2 Longo-Mbenza (DR Congo)

3 Christensen (Kenya)

4 Nzambi (DR Congo)

5 Unwin (Tanzania)

6 Bovet (Seychelles)

7 Delisle (Benin)

8 Motala (South Africa)

9 Kruger (South Africa)

10 Oladapo-Lola (Nigeria)

11 Schutte (South Africa)

12 Njelekela (Tanzania)

13 Mollentze (South Africa)

14 Walsh (South Africa -Urban)

15 Walsh (South Africa -Rural)

16 Crowther (South Africa)

17 Durban Diabetes Study (South Africa)

1

2

3

4

5

6

7

8

9

10

11

12

13

14

15

16

17

Optimal cut-point in the

derivation dataset

70

80

90

100

110

Waist circumference (95% CI) (cm)

0

10

20

30

40

50

Prevalence of obesity (BMI=30+), %

Study-specific waist circumference cut-point

**Figure S5.** Study-specific waist circumference cut-points for predicting at least two of the other components of metabolic syndrome in women by prevalence of obesity
