# Supplementary material for: Deriving an optimal threshold of waist circumference for detecting cardiometabolic risk in sub-Saharan Africa
Source: Int J Obes (Lond). 2017 Oct 31;42(3):487–94. doi: 10.1038/ijo.2017.240 (PMC5880575; doi:10.1038/ijo.2017.240)
Supplement: Supplementary Figure 6 [file ijo2017240x13.docx]

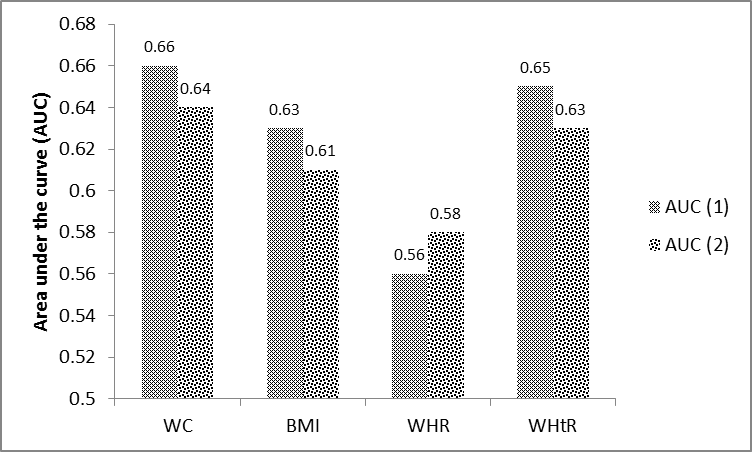


AUC (1) Area under curve from receiver operating characteristic (ROC ) curve analysis for predicting at least 2 of 4 components of metabolic syndrome excluding waist circumference

AUC (2) Area under the curve from receiver operating characteristic (ROC ) curve analysis for predicting at least 1 of 3 components of metabolic syndrome excluding waist circumference and low HDL

Abbreviations: AUC area under the curve; WC waist circumference (cm); BMI body mass index (Kg/m^2^); WHR waist hip ratio; WHtR waist-to-height ratio

**Figure S6.** The discriminatory power of waist circumference in predicting the presence of one component of metabolic syndrome including/excluding low HDL in men and women in the derivation dataset (Number of participants, 19 880**:** Men 8055, Women 11 825)
