# Supplementary material for: Deriving an optimal threshold of waist circumference for detecting cardiometabolic risk in sub-Saharan Africa
Source: Int J Obes (Lond). 2017 Oct 31;42(3):487–94. doi: 10.1038/ijo.2017.240 (PMC5880575; doi:10.1038/ijo.2017.240)
Supplement: Supplementary Figure 7 [file ijo2017240x14.docx]

Men

Women

0

20

40

60

80

Sensitivity (95% CI) (%)

15-19

25-29

35-39

45-49

55-59

65-69

75-79

85-89

Age (Years)

Abbreviations: CI confidence interval; WC waist circumference

**Figure S7.** Sensitivity of the derived waist circumference cut-point by age and sex in the derivation dataset (Number of participants, 19 880**:** Men 8055, Women 11 825)
