# Supplementary material for: Deriving an optimal threshold of waist circumference for detecting cardiometabolic risk in sub-Saharan Africa
Source: Int J Obes (Lond). 2017 Oct 31;42(3):487–94. doi: 10.1038/ijo.2017.240 (PMC5880575; doi:10.1038/ijo.2017.240)
Supplement: Supplementary Figure 8 [file ijo2017240x15.docx]

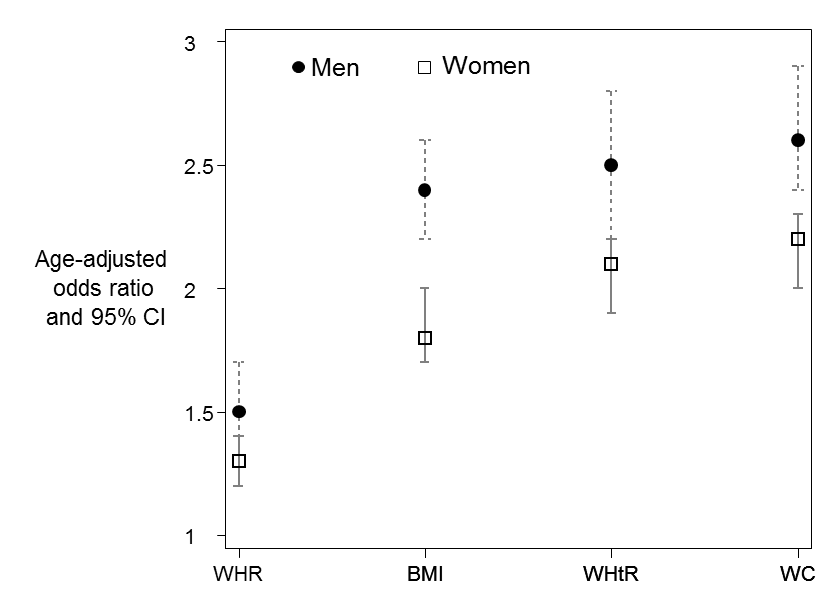


Abbreviations: CI confidence interval; WC waist circumference; BMI body mass index; WHR waist-hip ratio; WHtR weight to height ratio.

**Figure S8.** Age-adjusted odds ratio of having at least two components of metabolic syndrome comparing individuals classified as having raised marker of adiposity to individuals classified as normal in the derivation dataset (Number of participants, 19 880**:** Men 8055, Women 11 825)
